# Satellite repeat RNA expression in epithelial ovarian cancer associates with a tumor-immunosuppressive phenotype

**DOI:** 10.1172/JCI155931

**Published:** 2022-08-15

**Authors:** Rebecca L. Porter, Siyu Sun, Micayla N. Flores, Emily Berzolla, Eunae You, Ildiko E. Phillips, Neelima KC, Niyati Desai, Eric C. Tai, Annamaria Szabolcs, Evan R. Lang, Amaya Pankaj, Michael J. Raabe, Vishal Thapar, Katherine H. Xu, Linda T. Nieman, Daniel C. Rabe, David L. Kolin, Elizabeth H. Stover, David Pepin, Shannon L. Stott, Vikram Deshpande, Joyce F. Liu, Alexander Solovyov, Ursula A. Matulonis, Benjamin D. Greenbaum, David T. Ting

**Affiliations:** 1 Mass General Cancer Center, Harvard Medical School, Charlestown, Massachusetts, USA.; 2 Department of Medicine, Massachusetts General Hospital, Harvard Medical School, Boston, Massachusetts, USA.; 3 Division of Gynecologic Oncology, Dana-Farber Cancer Institute, Boston, Massachusetts, USA.; 4 Computational Oncology, Department of Epidemiology and Biostatistics, Memorial Sloan Kettering Cancer Center, New York, New York, USA.; 5 Department of Surgery, Massachusetts General Hospital,; 6 Department of Pathology, Brigham and Women’s Hospital, and; 7 Department of Pathology, Massachusetts General Hospital, Harvard Medical School, Boston, MA, USA.

**Keywords:** Oncology, Cancer, Innate immunity, Noncoding RNAs

## Abstract

Aberrant expression of viral-like repeat elements is a common feature of epithelial cancers, and the substantial diversity of repeat species provides a distinct view of the cancer transcriptome. Repeatome profiling across ovarian, pancreatic, and colorectal cell lines identifies distinct clustering independent of tissue origin that is seen with coding gene analysis. Deeper analysis of ovarian cancer cell lines demonstrated that human satellite II (HSATII) satellite repeat expression was highly associated with epithelial-mesenchymal transition (EMT) and anticorrelated with IFN-response genes indicative of a more aggressive phenotype. SATII expression — and its correlation with EMT and anticorrelation with IFN-response genes — was also found in ovarian cancer RNA-Seq data and was associated with significantly shorter survival in a second independent cohort of patients with ovarian cancer. Repeat RNAs were enriched in tumor-derived extracellular vesicles capable of stimulating monocyte-derived macrophages, demonstrating a mechanism that alters the tumor microenvironment with these viral-like sequences. Targeting of HSATII with antisense locked nucleic acids stimulated IFN response and induced MHC I expression in ovarian cancer cell lines, highlighting a potential strategy of modulating the repeatome to reestablish antitumor cell immune surveillance.

## Graphical abstract

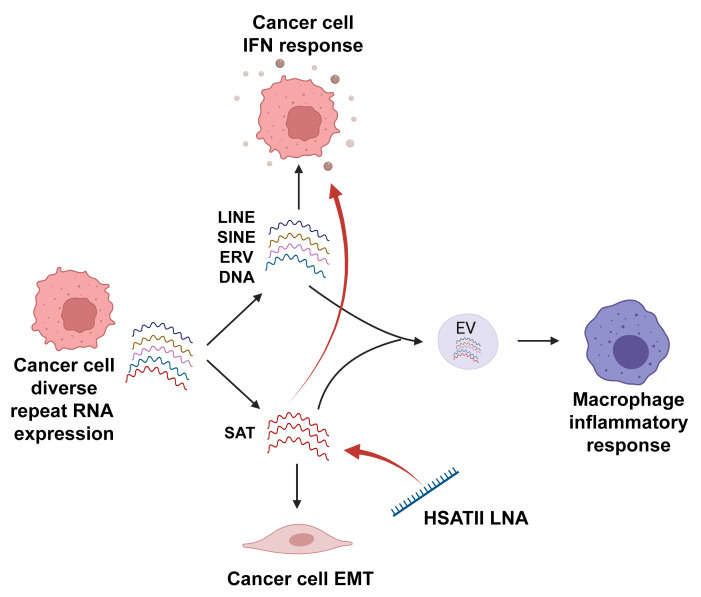

## Introduction

Repetitive elements make up approximately 50% of the human genome (1, 2), and their aberrant expression has been described across a wide range of cancers (3–5). Repeat suppression is achieved through a combination of epigenetic modifications (6) and the activity of tumor suppressors, including TP53 (7, 8). The loss or mutation of these guardians of the epigenome and genome leads to derepression of repeat RNAs that are sensed by pattern recognition receptors and trigger an innate immune system–mediated IFN response analogous to antiviral host response (8–16). However, the repeatome is diverse and exhibits differences in cellular and immunological response based on repeat RNA sequence motifs (17) and variation in coexpression of distinct clusters of repeats (5, 18, 19). In addition, some repeats (e.g., HERV) have been associated with response to immune checkpoint inhibitors (19–21), while other repeats are associated with immune-depleted tumor microenvironments (19, 22). Collectively, these studies link different cancer cell–intrinsic immune responses with expression of specific classes of repeat RNAs. To understand the relationship between repeat RNAs and immune response, we have focused on epithelial ovarian cancers (EOCs), given the role of TP53 in regulating repeat elements (7, 8) and the high rates of *TP53* mutations in EOC (23).

## Results

### Total RNA-Seq profiling reveals distinct clusters of repeat RNA expression across different epithelial cancers.

**Figure 1:**
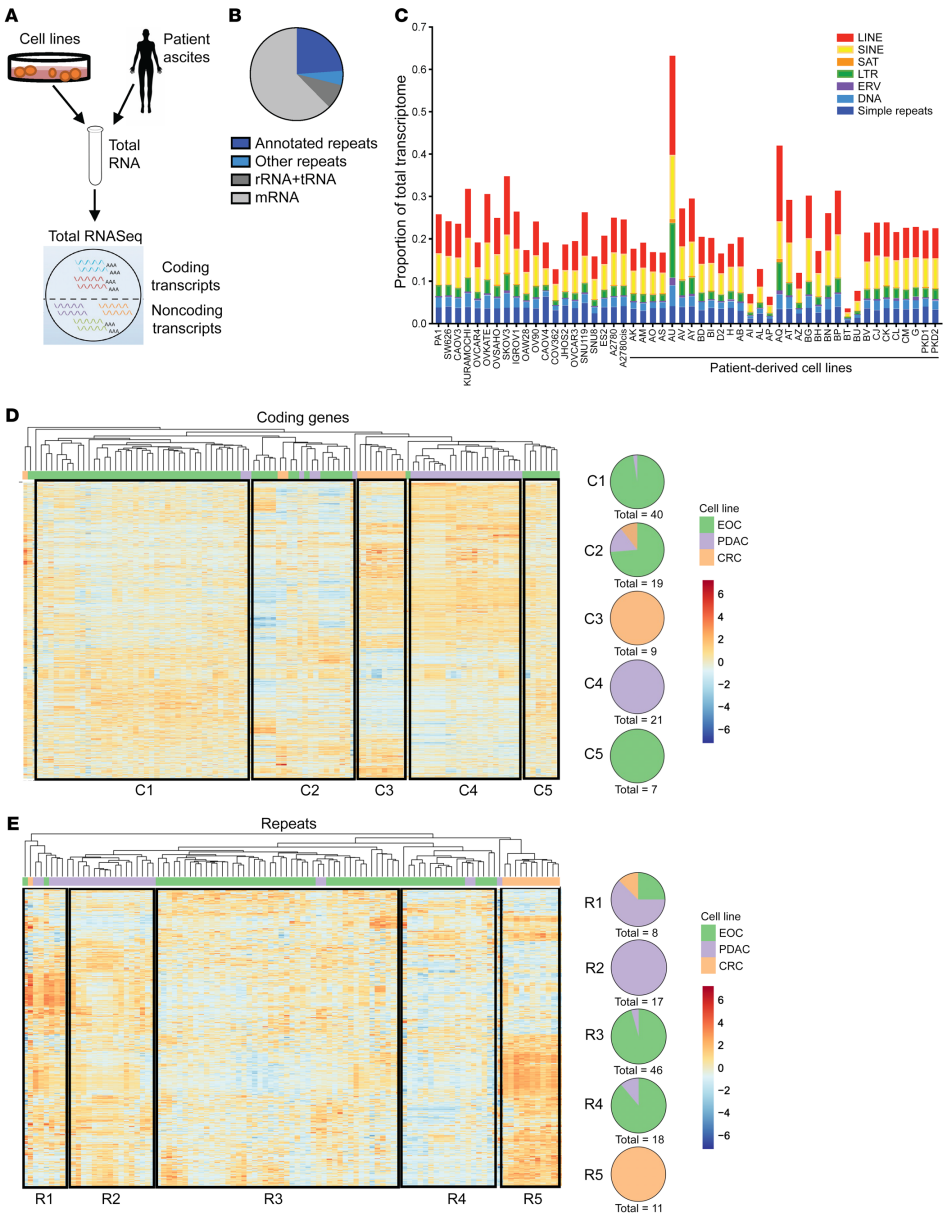
Diverse repeat RNA expression profiles are present in epithelial cancers and cluster tumors by tissue of origin distinctly compared with coding gene-based clustering. (**A**) Graphical abstract of experimental strategy. (**B**) Proportion of the total transcriptome represented by mRNA, ribosomal RNA/transfer RNA (rRNA/tRNA), annotated repeats, and nonannotated repeats, averaged across all epithelial ovarian cancer (EOC) cell lines. (**C**) Quantification of subclasses of repeat RNAs across EOC models using total RNA-Seq expressed as proportion of total transcription, including coding and noncoding reads in each cell line or in patient-derived cells. (**D**) Heatmap and hierarchical clustering of EOC (green), pancreatic ductal adenocarcinoma (PDAC; purple), and colorectal cancer (CRC; gold) cell lines by coding gene expression, including all coding genes that were differentially expressed between any 2 cell lines (adjusted *P* < 0.05 and |log_2_fold change| >1). Expression is plotted as scaled log_2_(normalized counts per million). Pie charts C1–C5 depict the cancer-type composition of each cluster as labeled. (**E**) Heatmap and hierarchical clustering of EOC (green), PDAC (purple), and CRC (gold) cell lines by repeat RNA expression, including all repeat species that were differentially expressed between any 2 cell lines (adjusted *P* < 0.05 and |log_2_fold change| >1). Expression is plotted as scaled log_2_(normalized counts per million). Major clusters defined by similar repeat expression profiles are outlined by black boxes. Pie charts R1–R5 depict the cancer-type composition of each cluster as labeled.

In order to comprehensively define the expression of repeat RNAs in EOC and compare this with expression in other cancers, we applied our previously established computational alignment methods for total RNA-Seq (19) to 31 patient-derived low-passage EOC, 17 commercially available ovarian, 26 pancreatic ductal adenocarcinoma (PDAC), and 11 colorectal cancer (CRC) cell lines (Figure 1A). In EOC models, this confirmed high contribution of noncoding transcripts to the total transcriptome (Figure 1B), with all major subclasses of repeats represented (Figure 1C). Expression levels of individual repeat RNAs varied, with some repeat RNAs (e.g., L1HS and HERVH) expressed at high levels that were comparable to those of traditional housekeeping genes, such as *ACTB* and *GAPDH* (Supplemental Figure 1A; supplemental material available online with this article; https://doi.org/10.1172/JCI155931DS1). Across EOC, PDAC and CRC, clustering of cell lines by coding genes segregated the samples by cancer type with high accuracy (Figure 1D). As expected, given unique transcriptional programs associated with different cancer types, major clusters comprising nearly entirely EOC (cluster 1 and cluster 5), CRC (cluster 3), and PDAC (cluster 4) cell lines emerged, with 1 additional cluster comprising samples of all 3 cancer types (cluster 2). Notably, clustering by repeat RNA expression alone was able to similarly distinguish between cancer types with few exceptions (Figure 1E), suggesting that, despite common overall repeat dysregulation across epithelial cancers, some repeat RNA species are cancer-type specific and may have important biological roles or consequences in these tumors.

### Satellite repeat RNAs cluster distinctly from other repeat elements and display variable expression across cancer models.

**Figure 2:**
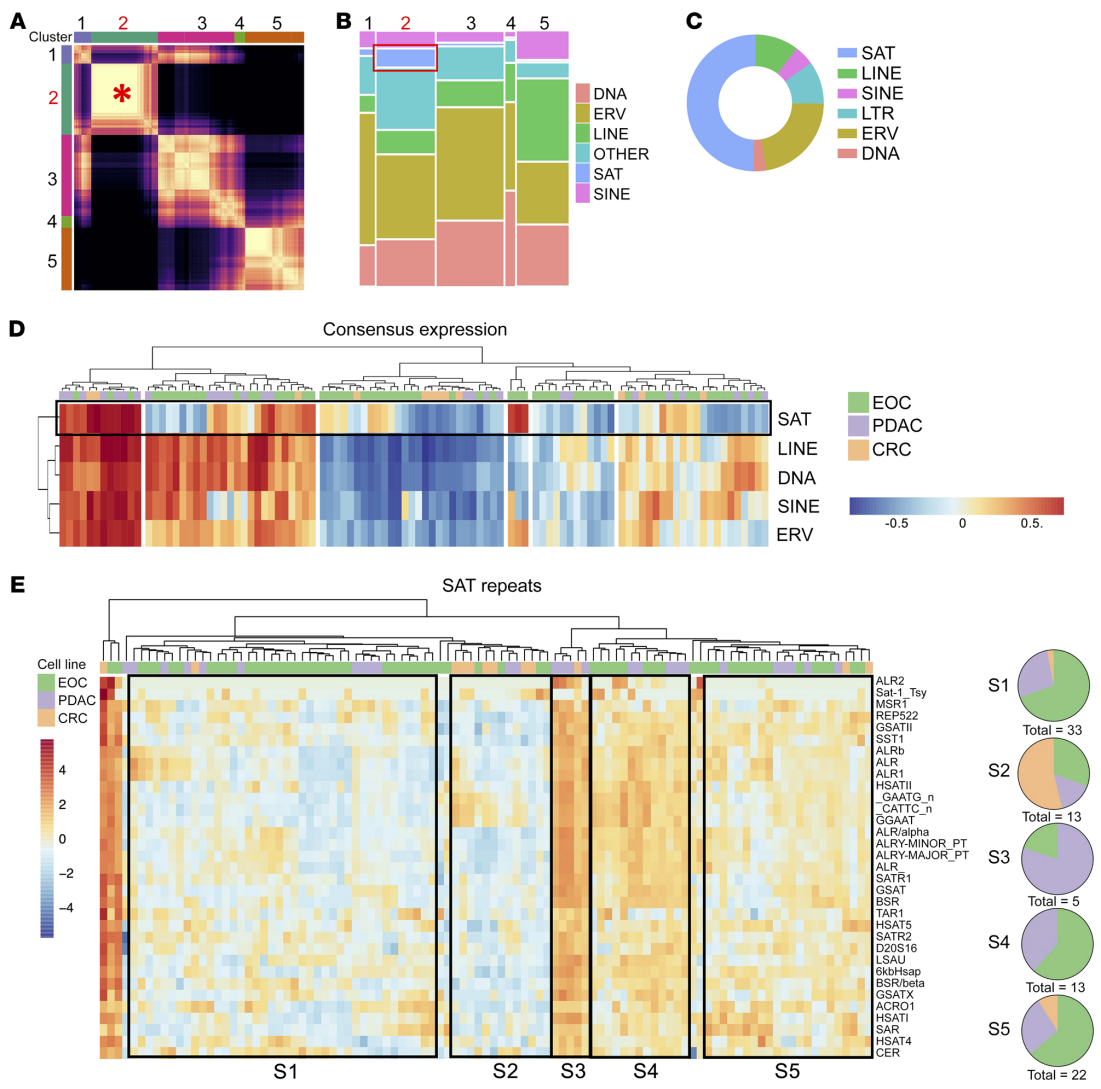
Repeat RNAs are coregulated in discrete clusters, with satellite repeat RNAs exhibiting unique expression patterns in epithelial cancers. (**A**) Heatmap for consensus clustering of repeat elements based on normalized expression. The red asterisk highlights satellite repeat–driven (SAT-driven) cluster 2, which has the strongest consensus correlation of the analyzed clusters. (**B**) Mosaic plot demonstrating relative repeat element subclass composition of each consensus cluster from **A.** The red box indicates SAT representation in cluster 2. (**C**) Proportion of total repeat expression for each subclass within the top 50 variant repeat RNAs across cell lines. (**D**) Hierarchical clustering of consensus expression of each repeat subclass across EOC (green), PDAC (purple), and CRC (gold) cell lines, depicting SAT consensus expression distinct from consensus expression of other repeat subclasses. (**E**) Heatmap and hierarchical clustering of EOC (green), PDAC (purple), and CRC (gold) cell lines by SAT RNA expression. Expression is plotted as scaled log_2_(normalized counts per million). Major clusters defined by similar SAT expression profiles are outlined by black boxes. Pie charts S1–S5 depict the cancer-type composition of each cluster, highlighting clusters distinct from tissue of origin.

To identify subclasses of repeat RNAs with biological relevance across tissue types, consensus clustering analysis of repetitive elements across all cell line samples was performed (see Methods). We detected 5 distinct clusters of coexpressed repetitive elements within cluster 2, demonstrating the strongest consensus correlation across samples (Figure 2A, denoted by the red asterisk, and Supplemental Figure 1B). Subclass analysis revealed an enrichment for satellite (SAT) repeats in cluster 2 (Figure 2B). Notably, SAT expression was found to be highly variable in EOC cell lines, with SAT RNAs representing the highest proportion of the 50 most variant transcripts (Figure 2C). In line with this, clustering of consensus expression profiles for each major subclass of repeats showed the distinct expression patterns of the SAT subclass across cell lines (Figure 2D). Furthermore, hierarchical clustering of EOC, PDAC, and CRC cell lines based on SAT RNA expression demonstrated unique clustering of samples that was not driven by tissue of origin (Figure 2E). This indicated that SAT RNA expression patterns could have shared transcriptional programs across diverse epithelial cancers.

### SAT repeat RNA expression is linked with an immunosuppressive epithelial-mesenchymal transition gene expression pattern in EOC.

**Figure 3:**
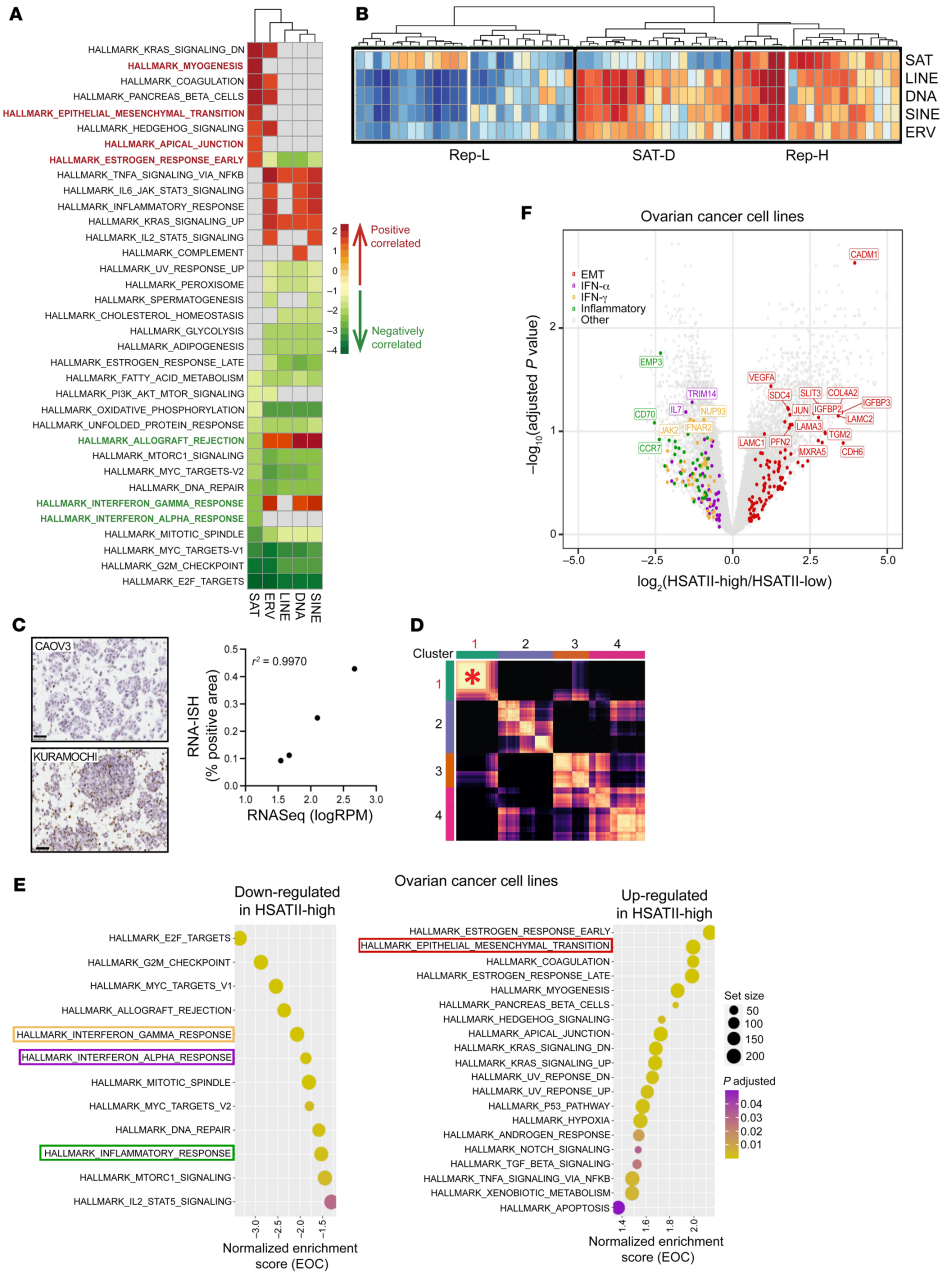
Satellite repeat expression is associated with upregulation of epithelial-mesenchymal transition and downregulation of innate immune-response genes in EOC models. (**A**) Heatmap of enriched Gene Ontology terms identified using gene set enrichment analysis (GSEA) plotted based on normalized enrichment score. GSEA was applied to a ranked gene list based on correlation with the consensus expression calculated for each repeat subclass, with the FDR set at 0.05. Positive enrichment scores (red) indicate functions that positively correlate with repeat subclass expression. Negative enrichment scores (green) indicate functions that negatively correlate with repeat expression. (**B**) Hierarchical clustering of consensus expression calculated for each repeat subclass in EOC cell lines. Major clusters are outlined by black boxes. (**C**) Representative RNA-ISH images with HSATII-specific probes in 2 EOC cell lines and correlation (Pearson’s *r*^2^) between HSATII RNA expression as determined with RNA-Seq by log(reads per million[RPM]) (as determined with RNA-Seq, with log(reads per million) as units) and percentage of tumor cells with a positive staining for HSATII by RNA-ISH. Original magnification, ×40 (**D**) Heatmap for consensus clustering of all repeat elements except HSATII, which was removed from analysis, based on normalized expression. The asterisk highlights SAT-driven cluster 1, which shows the highest consensus correlation of analyzed clusters, similar to clustering when HSATII was included. (**E**) GSEA of hallmark terms ranked on the basis log_2_FC of coding genes for pathways containing genes that are upregulated and downregulated in HSATII-high compared with HSATII-low ovarian cancer cell lines, based on highest (Q4) and lowest (Q1) quantile (see Supplemental Figure 3C). Colored boxes represent the pathways indicated in **F**. Circle size represents gene set size, and circle color represents adjusted *P* value. (**F**) Volcano plot depicting differentially expressed coding genes between HSATII-high and HSATII-low EOC cell lines. EMT, IFN-α, IFN-γ, and inflammatory hallmark pathways are highlighted.

To better characterize the relationship of repeatome profiles with coding gene behavior in EOC, we first applied gene set enrichment analysis (GSEA) with the hallmark gene set from the Broad Institute’s Molecular Signatures Database (24, 25) to a gene list ranked based on correlation with the consensus expression calculated for each repeat subclass across EOC cell lines. This demonstrated high positive correlation of SAT repeats with the epithelial-mesenchymal transition (EMT) gene set and anticorrelation with several immune and IFN-response sets, including the IFN-α and IFN-γ gene sets (Figure 3A). A parallel analysis separating EOC cell lines into SAT-high and SAT-low cell lines on the basis of median consensus expression identified enrichment for the hallmark EMT gene set in SAT-high cell lines, while IFN-α, IFN-γ, and inflammatory-response gene sets were enriched in SAT-low cell lines (Supplemental Figure 2A), further validating these associations.

To further investigate this observation, hierarchical clustering of EOC models by consensus expression of repeat RNA subclasses was performed; this separated EOC cell lines into 3 major clusters, as depicted in Figure 3B. Repeat-high (Rep-H) cell lines displayed high expression of all subclasses, while repeat-low (Rep-L) cell lines had relatively low repeat RNA expression in general. A third distinct cluster also emerged; it exhibited high expression of all subclasses of repeat RNAs except for SAT RNAs, which we referred to as SAT-depleted (SAT-D) cell lines. To further characterize the specific contribution of SAT repeats specifically, GSEA was performed on Rep-H and SAT-D EOC cell lines (Supplemental Figure 2B). GSEA demonstrated enrichment of EMT-related genes and downregulation of genes related to innate immune and IFN-response pathways in the Rep-H cell lines compared with SAT-D cell lines, confirming the association observed in the total cohort when analyzed by correlation with SAT expression (Figure 3A and Supplemental Figure 2A). Rep-L EOC had higher enrichment of cell cycle– and replication-related pathways (hallmark E2F, G2M, MYC targets), indicating an anticorrelation of repeat expression with mitotic activity. Collectively, this refined repeat subtyping identifies unique characteristics, including high EMT expression in Rep-H cell lines, activation of IFN-response genes in SAT-D cells lines, and high proliferative activity in Rep-L cells lines.

### Human SAT II is a representative SAT repeat RNA that correlates with worsened clinical outcomes in human EOC.

In order to further investigate the biological implications of high SAT expression in EOC, we selected human SAT II (HSATII) as a representative repeat species within the SAT subclass; we had previously found it enriched across epithelial cancers (3). As expected, HSATII expression was significantly higher in Rep-H cell lines (Supplemental Figure 3A); this was validated in a subset of cell lines by RNA-ISH (Figure 3C). In EOC samples in which HSATII has been removed, consensus clustering analysis of repetitive elements across samples (Figure 3D and Supplemental Figure 3B) yielded a similar SAT-driven cluster that displayed the strongest consensus correlation; this implies that HSATII is not the sole driver of the SAT subclass but is instead a representative member. Differential gene expression analysis was run on HSATII-low and HSATII-high EOC cell lines to determine coding gene expression patterns linked with HSATII expression. This revealed that genes related to EMT were upregulated and genes related to IFN-response and inflammatory pathways were downregulated in HSATII-high samples (Figure 3, E and F). These results are similar to those from the same comparisons based on total SAT expression (Supplemental Figure 2A).

**Figure 4:**
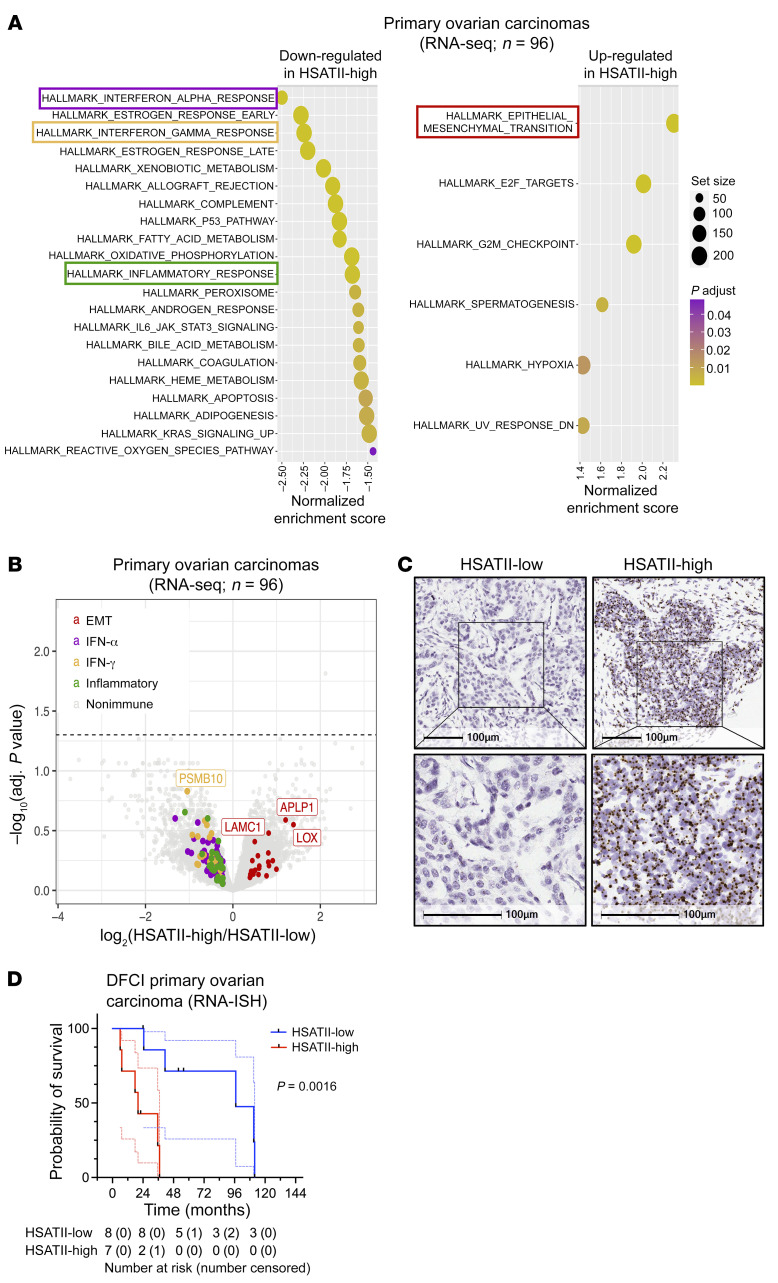
High satellite repeat expression is linked with upregulation of epithelial-mesenchymal transition, suppressed immune response, and worsened clinical outcomes in primary human EOC. (**A**) GSEA results ranked by normalized enrichment score for pathways containing genes that are upregulated (right) and downregulated (left) in HSATII-high compared with HSATII-low early-stage human ovarian carcinoma samples (*n =* 96). Colored boxes represent the pathways indicated in **B**. Circle size represents gene set size, and circle color represents adjusted *P* value. (**B**) Volcano plot depicting differentially expressed coding genes between HSATII-high and HSATII-low early-stage human ovarian carcinoma samples (*n =* 96). Genes driving the enrichment of EMT, IFN-α, IFN-γ, and inflammatory Hallmark pathways are highlighted. (**C**) Representative images of RNA-ISH with an HSATII-specific probe, depicting an example of an HSATII-low (left) and HSATII-high (right) primary human EOC tumor. Scale bar: 100 μm. (**D**) Kaplan-Meier survival curves for HSATII-high (red) and HSATII-low (blue) in a cohort of 16 primary human EOC tumors using quantified RNA-ISH. All data points and 95% CI are shown (dotted lines). Number at risk is number of patients in the analysis at that time point. Number censored are those who did not experience an event but had their last data point at that time interval. Log-rank, *P* = 0.0016.

To interrogate the association of SAT repeats with transcriptional programs in patients, we investigated patterns of HSATII expression in total RNA-Seq data from a cohort of 96 human primary ovarian carcinomas (26). Similar to EOC cell lines, tumors with high levels of HSATII expression (Supplemental Figure 3C) demonstrated upregulation of genes related to EMT and downregulation of genes in the IFN-α, IFN-γ, and inflammatory pathways (Figure 4, A and B) compared with those with low HSATII expression. We then performed HSATII RNA-ISH with quantitative image analysis in a separate cohort of patients with advanced high-grade serous ovarian cancer (HGSOC) from the Dana-Farber Cancer Institute to segregate primary tumors into those with low or high HSATII expression (Figure 4C). Notably, separating tumors by HSATII expression revealed significantly shorter overall survival of patients with HSATII-high tumors (Figure 4D). Taken together, this work shows that repeats are a diverse set of RNA species; some are associated with tumor cell IFN response (9, 10, 12, 13, 27, 28), while others, such as SAT repeats, are associated with EMT and low IFN signaling that is typically seen in more aggressive tumors.

**Figure 5:**
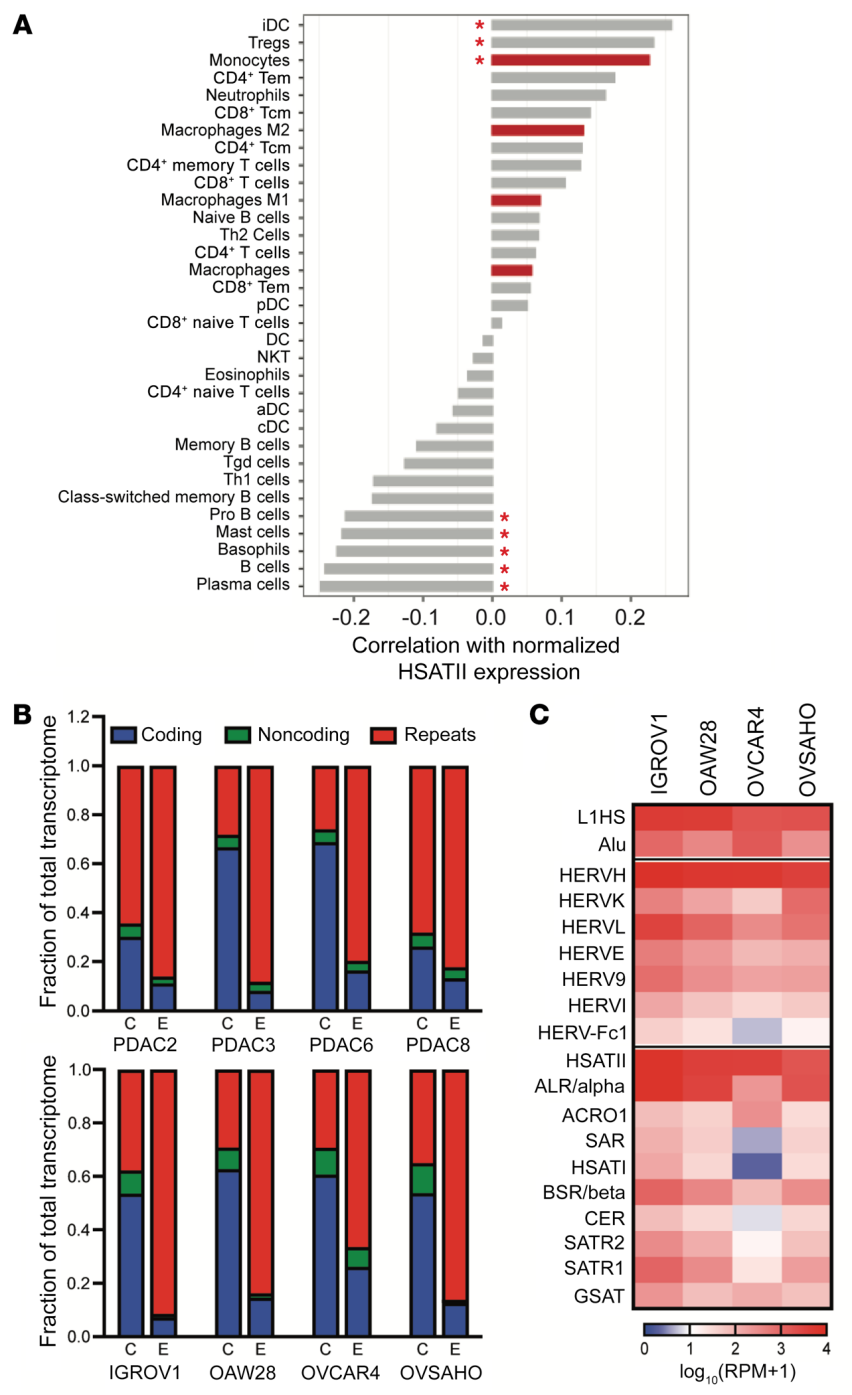
Repeat RNAs enriched in tumor cell–derived extracellular vesicles can induce changes in the tumor immune microenvironment. (**A**) Pearson’s correlation coefficients between normalized HSATII expression and the relative frequency of immune cell types in 96 human early-stage ovarian carcinoma tumor samples as identified by the xCell algorithm. Red asterisks indicate correlations with *Q* < 0.1. *P < 0.05. (**B**) RNA content of tumor cells (C) and tumor cell–derived extracellular vesicles (E) in PDAC (top) and EOC (bottom) cell lines, as determined by total RNA-Seq and plotted as a fraction of the total transcriptome. (**C**) Expression heatmap of representative repetitive elements in extracellular vesicles released by EOC cell lines.

**Figure 6:**
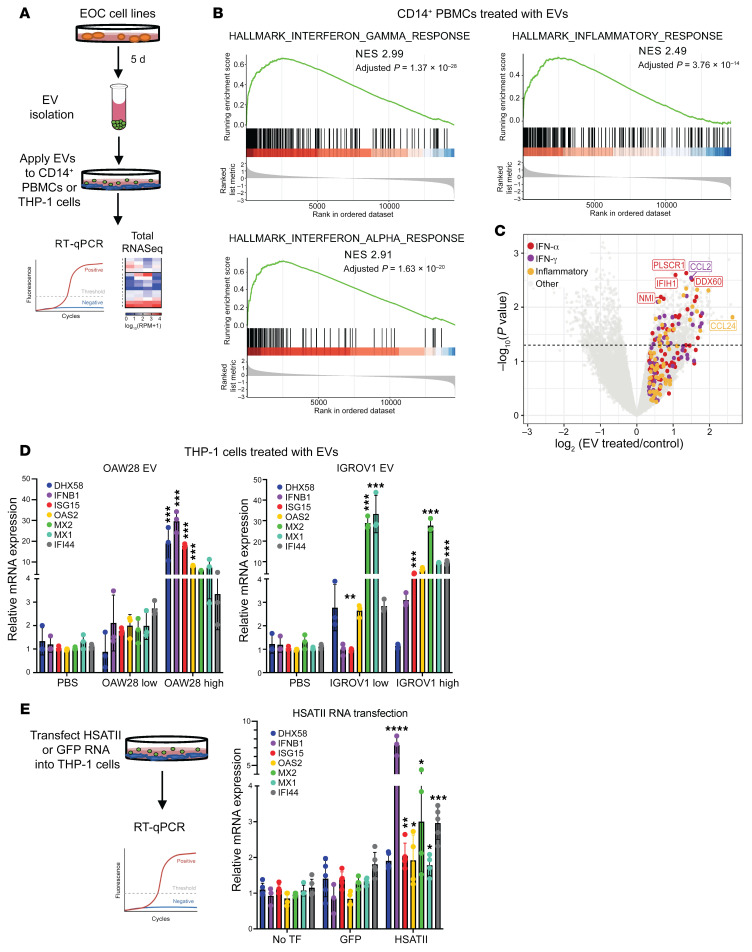
Repeat RNA-enriched extracellular vesicles can induce changes in the tumor immune microenvironment. (**A**) Schema of experimental design relating to data in **B**–**E**. (**B**) Gene set enrichment analysis of IFN-response signatures and inflammatory response in extracellular vesicle–treated (EV-treated) versus untreated samples. NES, normalized enrichment score. (**C**) Volcano plot depicting the differential expression of coding genes between EV-treated and untreated EOC cell lines. Genes driving the enrichment in IFN-α, IFN-γ, and inflammatory hallmark pathways are noted. (**D**) Quantitative RT-PCR of IFN-response genes from THP-1 monocyte cell line treated with high-dose or low-dose EVs from ovarian cell lines, OAW28 (left) and IGROV1 (right). (**E**) Schema of THP-1 cells treated with HSATII or GFP RNA transfection and quantitative RT-PCR of IFN-response genes without transfection (TF) or with transfection of GFP RNA or HSATII RNA. For RT-PCR, all data points are shown as mean ± SD. One-way ANOVA analysis was performed with Tukey’s multiple comparisons test; significance is shown between EV treatment and PBS or HSATII and GFP RNA. *P < 0.05, **P < 0.01, ***P < 0.001, ****P < 0.0001.

Given the correlation of HSATII with low IFN response, high EMT, and worsened survival, we next evaluated the relationship between HSATII expression and the immune microenvironment in ovarian cancer. Cellular deconvolution analysis of the 96 total RNA-Seq ovarian tumors using xCell (29) was performed to estimate percentages of specific immune populations and then calculate their correlation with HSATII expression (Figure 5A and Supplemental Figure 4A). Immune cells positively correlated with HSATII included immature dendritic cells, Tregs, and myeloid cells (monocytes, macrophages, neutrophils), indicating an immune microenvironment dominated by innate immune cells. Given our prior work demonstrating that some noncoding RNAs expressed in cancer cells can directly activate cells of the mononuclear phagocytic system (17), we hypothesized that extracellular vesicles (EVs) could serve as a vehicle to deliver HSATII and other repeats with the ability to modulate innate immune cells within the tumor microenvironment. To test this, we first collected EVs released by PDAC and EOC cell lines and confirmed that isolated EVs expressed typical EV-associated cell surface markers (Supplemental Figure 4B). RNA was then purified from tumor cell–derived EVs and subjected to total RNA-Seq. Compared with the RNA profile of each parental cell line, a robust enrichment of a diverse set of repeat RNAs was detected in EVs isolated from each cell line (Figure 5B), with HSATII being one of the most prevalent RNAs (Figure 5C). To then test the effect of HSATII-enriched EVs on human myeloid cells, we purified EOC-derived EVs and applied them to flow cytometry–sorted CD14^+^ PBMCs collected from healthy human donors (Figure 6A). CD14^+^ PBMCs exposed to EOC EVs demonstrated upregulation of genes related to the activation of the innate immune and IFN responses (hallmark IFN-α, IFN-γ, and inflammatory response) compared with unexposed CD14^+^ cells (Figure 6, B and C). A similar activation of genes within these pathways was observed in response to both PDAC and EOC tumor cell–derived EVs and in CD14^+^ cells from multiple individual healthy donors in separate experiments (Supplemental Figure 4C), suggesting a common response of monocyte-derived cells to repeat RNA–enriched EVs in the tumor microenvironment.

Given the presence of multiple classes of repeat RNAs in EOC-derived EVs (Figure 5B) and the distinct effects on tumor and immune cells conferred by different repeat RNA species, we sought to determine if HSATII specifically can stimulate myeloid cells. To test this, we used THP-1 monocytic cells to evaluate the responses of these innate immune cells to treatment with EOC EVs and in vitro transcribed HSATII RNA. THP-1 cells treated with EOC EVs from 2 different cell lines had significant induction of IFN-response genes, including *DHX58*, *IFNB1*, *ISG15*, *OAS2*, *MX1*, *MX2*, and *IFI44*, as measured by quantitative RT-PCR (Figure 6D). HSATII transfection, compared with GFP RNA transfection, significantly induced expression of *IFNB1*, *OAS2*, *ISG15*, *MX1*, *MX2*, and *IFI44*, which indicates that HSATII RNA in EVs partially contributes to the IFN response seen by EOC EVs in monocyte-derived cells (Figure 6E). This suggests that HSATII is sensed by and can generate an IFN response in immune cells that are enriched in the tumor microenvironment of HSATII-high tumors.

### Modulating the repeatome with epigenetic drugs or repeat-specific antisense oligos has diverse effects in EOC.

We have shown that repeat RNAs can be transmitted to responding innate immune cells and drive an IFN response. However, our collective analyses in EOC cell line models and tumors has indicated that tumor cells with high baseline levels of SAT RNAs lack IFN pathway activation, implying that they have developed an adaptation to suppress the IFN response to repeats. Therefore, we hypothesized that modulating different repeats in tumor cells may overcome this repeatome tolerance. Repetitive elements are known to be suppressed in the normal genome, in part, by DNA and histone methylation (7, 8), and epigenetic therapies have been shown to induce transcription of some repeat species in ovarian cancer models (9, 12, 13). Thus, we first tested the effect of treatment with a DNA methyltransferase inhibitor (DNMTi; 5-azacytidine, 500 nM) and a histone deacetylase inhibitor (HDACi; trichostatin A, 250 nM) on EOC cell lines. As expected, these drugs induced broad changes in repeat element expression, but there were notable differences, with DNMTi promoting a greater induction of ERV, SINE, and LINE elements, while HDACi consistently increased SAT elements across cell lines (Supplemental Figure 5, A and B). Analysis of coding genes induced by these agents revealed enrichment of IFN-response gene expression in cell lines treated with DNMTi, whereas EMT pathway genes were enriched in cell lines treated with HDACi (Supplemental Figure 5, C and D); this was consistent with coexpression patterns of these distinct repeat subsets in our EOC cell lines (Figure 3A) and tumors (Figure 4, A and B). These findings suggest that DNA methylation and histone acetylation have different contributions to the regulation of the repeatome profile in EOC, and, importantly, the response to these drugs can have discordant pro- and antitumoral effects on cancer cells.

**Figure 7:**
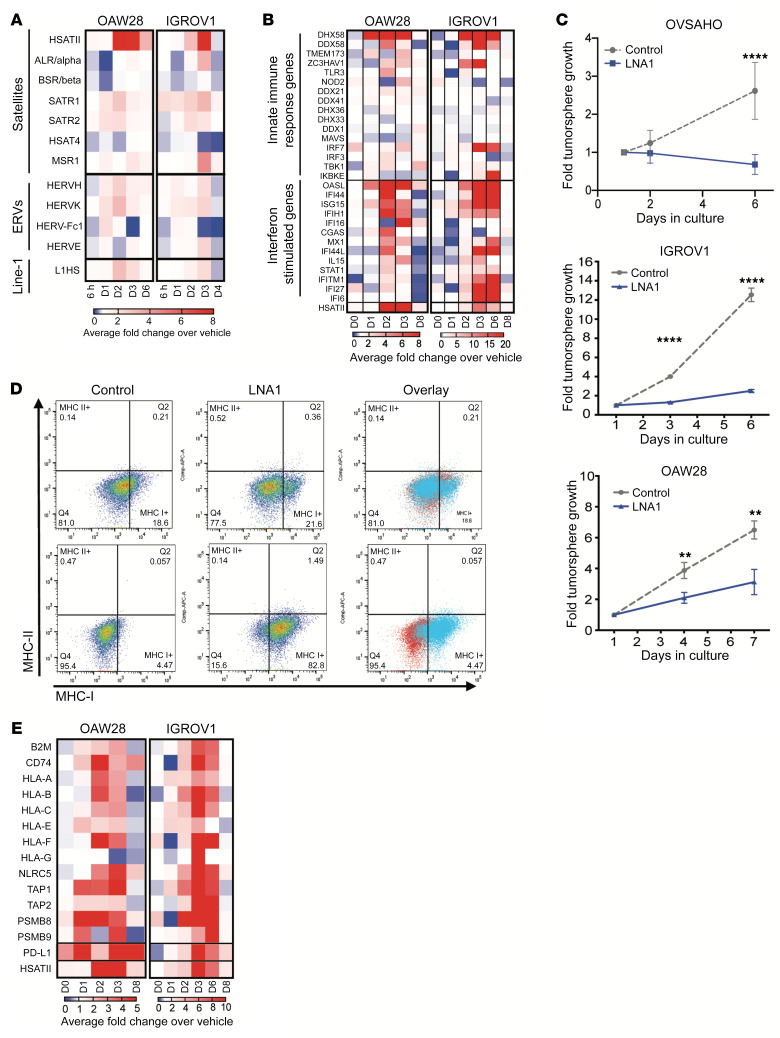
Modulation of HSATII RNA with LNA is cytotoxic, induces IFN response, and increases MHC class I expression. (**A**) Expression levels of HSATII and other repeat RNAs in EOC cell lines transfected with HSATII-specific LNA relative to scramble control LNA over time, plotted as fold change over control on days 0 through 6 after transfection. (**B**) Expression heatmap depicting relative expression of innate immune-response genes and IFN-stimulated genes (ISGs) in EOC cell lines transfected with HSATII-specific LNA relative to scramble control LNA over time. (**C**) Effect of HSATII-specific LNA (LNA1) on tumorsphere growth in EOC cell lines, as determined by 3D CellTiter-Glo viability assays. Plots represent 4 separate experiments for each cell line, with 2-tailed unpaired *t* test performed at each time point. **P < 0.01, ****P < 0.0001. (**D**) Flow cytometric analysis of MHC-I and MHC-II cell surface protein expression on EOC cell lines transfected with LNA1 compared with control LNA. (**E**) Expression heatmap depicting relative expression of MHC class I genes and PD-L1 in EOC cell lines transfected with HSATII-specific LNA relative to scramble control LNA over time.

Given the consistent relationship between SAT repeat expression and EMT-high and IFN-low phenotypes, we pursued direct targeting of the HSATII-specific locked nucleic acids (LNAs) as an antisense oligo therapeutic. HSATII LNAs and control scramble LNAs were transfected into EOC cell lines, followed by total RNA-Seq analysis at various times after transfection. This revealed a specific and marked increase in HSATII RNA in the cells, which peaked on days 2–3 (Figure 7A), with minimal off-target effects on other repeat RNA species. Analysis of the coding gene transcripts in HSATII LNA–transfected cells over time revealed an upregulation of innate immune-response genes and IFN-stimulated genes, indicating that HSATII LNAs could target cancer-specific HSATII RNA and trigger an IFN response (Figure 7B). In addition, EOC cells grown in nonadherent culture following HSATII LNA transfection consistently demonstrated a significant reduction in tumorsphere growth and, in the case of OVSAHO cells, increased cell death, compared with cells transfected with control LNA (Figure 7C). Further investigation into the immune-related transcriptional changes in HSATII LNA–transfected tumor cells also revealed alterations in expression of genes related to MHC class I (MHC-I) antigen presentation. Similar to the anticorrelation observed between steady-state HSATII levels and innate immune and IFN-response genes, we found that EOC cell lines (Supplemental Figure 5E) and primary human EOC tumors (Supplemental Figure 5F) with higher baseline HSATII RNA levels had decreased expression of MHC-I–related genes. However, EOC cells transfected with HSATII LNA revealed a striking upregulation of these MHC-I–related genes (Figure 7E). Furthermore, HSATII LNA–transfected EOC cell lines also demonstrated an increase in MHC-I proteins on the cell surface compared with control LNA-transfected cells (Figure 7D). Taken together, the increase in HSATII RNA levels by targeted LNA induced an IFN response associated with EOC tumor cell cytotoxicity and upregulation of MHC genes, suggesting the possibility that HSATII RNA modulation could sensitize EOC tumor cells to immunotherapy strategies.

## Discussion

Repeat RNAs are commonly expressed in EOC, yet despite their immunogenic potential, immune checkpoint inhibitors demonstrate only modest activity in EOC (30–33). This apparent challenge reflects the marked diversity of repeat RNA species that constitute the repeatome. Here, we demonstrated that various repeat species are coregulated in distinct clusters. These clusters exhibit diverse expression patterns across epithelial cancers and likely reflect the inherent differences in both tissue of origin and the genetic mutation background of each tumor. Furthermore, consensus clustering of repeat RNAs revealed a distinct pattern of SAT RNA coexpression as well as a high level of variation in SAT expression levels across EOC samples. Given this, we honed in on SAT repeats in EOC and strove to determine their patterns of expression and the tumor cell phenotypes and tumor microenvironmental characteristics associated with these patterns.

While some repeat species, such as ERVs and LINE elements, can activate IFN signaling (9, 12, 13) and SAT repeats are able to stimulate immune cells (17), this work revealed lower tumor cell–intrinsic IFN pathway activation in EOC models with high SAT expression. This suggests that tumor cells with high steady-state SAT repeat expression have the ability to tolerate this through suppression of IFN activation mechanisms, which merit further investigation. Conversely, this work also unveiled the striking link between expression of SAT repeats, including HSATII, and an EMT transcriptional program in tumor cells. This is consistent with mounting evidence that the EMT state in tumor cells is characterized by an immunosuppressed phenotype (34). Furthermore, HSATII-high cell lines exhibited suppressed levels of MHC-I gene expression, a phenomenon also linked to the EMT state of a cell (35, 36). In murine mammary carcinoma models, epithelial cells have high levels of MHC-I expression, whereas mesenchymal cells express low levels of MHC-I (35). This relationship was also observed in lung cancer cell lines, in which metastatic lines that had undergone EMT had lower expression of MHC-I genes than nonmetastatic cell lines (36).

This work suggests that EMT activation in SAT-expressing cancers likely promotes a tumor cell–intrinsic immunosuppression, possibly creating a permissive state in which tumor cells are tolerant of high levels of immunogenic repeats and resistant to immunotherapy. Indeed, prior transcriptional profiling of EOC has identified 4 molecular subtypes, including mesenchymal, immunoreactive, proliferative, and differentiated subtypes, with worsened overall survival observed in the mesenchymal subtype (37). Along with this association, higher HSATII levels in primary ovarian tumors were found to be correlated with worse outcomes in a cohort of patients with ovarian cancer; this is consistent with our prior observations linking increased HSATII copy numbers with lower survival rates in CRC (38).

**Figure 8:**
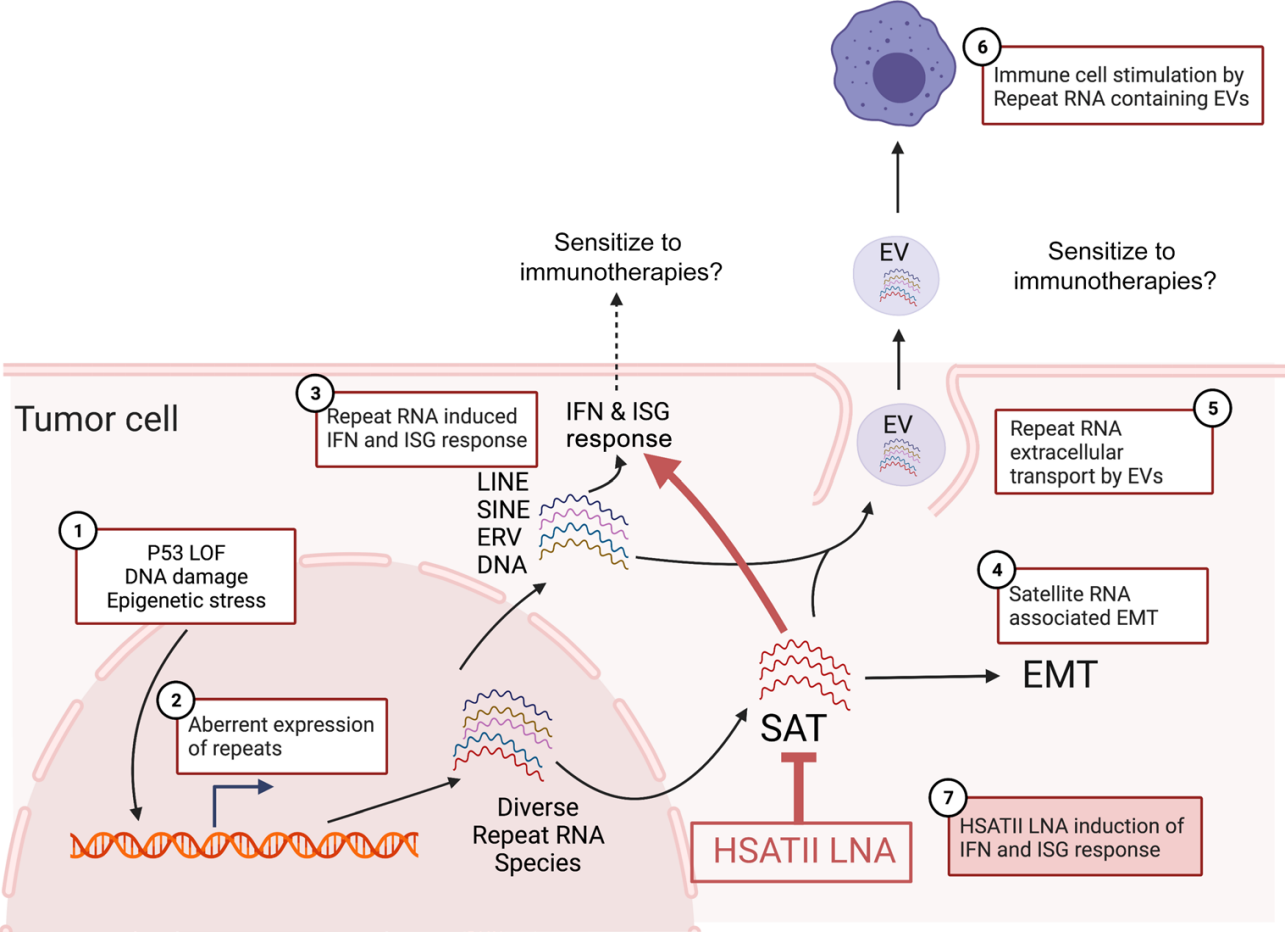
Working model of potential biologic and therapeutic implications of SAT RNA expression in epithelial tumor cells. Working model depicting the reported and hypothesized tumor cell autonomous and tumor microenvironmental effects of aberrant SAT RNA expression. Various genetic and/or epigenetic alterations (1) can lead to aberrant expression of repeat RNAs (2) in cancer cells. Certain subclasses of repeat RNAs can trigger an IFN response (3) and may sensitize tumors to immunotherapies. Other repeat species, like SAT, can preferentially stimulate EMT (4). Repeat RNAs can also leave the cell in exosomes (5) and thereby stimulate immune cells in the tumor microenvironment (6), which may also sensitize to immunotherapies. These pathways can be modulated by applying LNA to specifically target SAT RNA species like HSATII (7). This model highlights the potential utility of SAT repeat RNAs as biomarkers and therapeutic targets in EOC. LOF, loss of function.

Interestingly, a positive association with the presence of innate immune cells in primary tumor RNA-Seq profiles was revealed in HSATII-high models. In particular, monocytes, macrophages, and immature dendritic cells were enriched in HSATII-high ovarian cancers, suggesting a potential functional relationship between HSATII and these innate immune cells in the tumor microenvironment. Similarly, a relationship between EMT and immunosuppressive tumor–associated macrophages has been reported in many cancers, including EOC (34, 39). Our prior work shows that HSATII uniquely displays pathogen-associated CpG motifs and that monocyte-derived DCs and bone marrow–derived macrophages can be stimulated with transfection of HSATII RNA in a CpG-dependent manner (17). Taken together, this suggests a potential role for SAT RNAs in establishing or maintaining an immunosuppressive tumor microenvironment in EOC. While further mechanistic studies are required to draw this conclusion, our current findings that repeat RNA-enriched tumor-derived EVs can induce IFN-response genes in human primary monocytic cells and a macrophage cell line suggest a direct mechanism for repeat RNA modulation of the tumor immune microenvironment. Although these EVs contain many different repeat RNA species that may have the ability to stimulate IFN responses, including ERVs, we specifically demonstrated the ability of HSATII RNA to directly stimulate IFN-response genes in macrophages. This implies a distinct role for HSATII in modulating the tumor immune microenvironment. This is complementary to prior work showing HSATII enrichment in pancreatic cancer EVs (40) and more recent work demonstrating an association of repeat RNA expression with IFN response in fibroblasts in pancreatic cancer (22). A similar link between repeat-enriched EVs and alterations in the immune microenvironment of Ewing’s sarcoma has also been reported (41). Altogether, these collective studies imply that specific repeat RNA species are able to induce distinct transcriptional responses in tumor and microenvironmental cells. While SAT repeat expression leads to EMT and an immunosuppressed phenotype in tumor cells, when released outside of the tumor cell they can induce a secondary IFN response in myeloid cells that generates a tumor-permissive microenvironment (Figure 8). The strong correlation between HSATII RNA levels and clinical outcomes readily apparent in even a small cohort of patients with ovarian cancer from the Dana-Farber Cancer Institute also highlights the potential of repeat RNA species as prognostic biomarkers in ovarian and likely other cancers.

Beyond this, our work also highlights the potential therapeutic opportunities associated with modulation of repeat RNAs from a tumor cell–intrinsic perspective. Although HSATII-high cell lines were found to exhibit a transcriptional profile consistent with suppression of the innate immune and IFN-response pathways, we found that acutely elevating levels of HSATII RNA with HSATII-specific LNAs in the cell can overcome this suppression and induce significant cytotoxicity. While SAT RNAs were also upregulated in EOC cell lines treated with HDACi, this method of manipulation did not result in concomitant IFN pathway activation. This is likely a reflection of the HSATII specificity of an LNA compared with the broad effects of HDACi on SAT RNAs and other coding and noncoding RNAs. We note that TSA is a generalized HDACi and more specific HDAC class I and class II inhibitors merit further investigation. Interestingly, most LNAs are thought to decrease target RNA through RNase H–mediated degradation; however, our particular LNA design led to highly specific and robust elevation of HSATII RNA, presumably through stabilization of HSATII RNA species and inhibition of reverse transcriptional machinery (38) or other undetermined repeat RNA processing proteins. In addition, HSATII perturbation was found to result in upregulation of MHC-I genes and PD-L1 on tumor cells. Given that lack of MHC-I expression is a common mechanism of resistance to immune checkpoint blockade (42–44), these findings raise the possibility that, in an in vivo setting, SAT repeat RNA modulation could synergize with immune checkpoint blockade by resensitizing cells to anti–PD-1 agents (Figure 8); this is similar to other strategies to reexpress MHC-I in tumor models (45). This is particularly intriguing in EOC, a disease that is known to have cytotoxic T lymphocytes present in the tumor (46) while displaying very low response rates to single-agent checkpoint inhibitors (30, 31, 33, 47). Thus, strategies to render EOC tumors more susceptible to immune checkpoint blockade while simultaneously limiting immune-related toxicities are critically needed, and further investigation of repeat RNA modulation is warranted. Moreover, the demonstrated presence of HSATII and other SAT RNAs in tumor cell–derived EVs indicates the potential of SAT RNAs to be developed as blood-based prognostic and predictive biomarkers in EOC. Overall, in the context of growing interest in nucleic acid therapeutic technologies (48), our current findings highlight the potential to translate improved understanding of repeat element dysregulation in cancer to clinical use, particularly in cancers in which immunotherapy has proven only modestly effective.

## Methods

### Cell lines

#### EOC cell lines.

CAOV-4, IGROV1, JHOS-4, OAW28, OV90, OVCAR4, OVCAR8, and OVKATE cells were gifts from Cyril Benes (Massachusetts General Hospital Cancer Center, Boston, Massachusetts, USA). PA-1, CAOV-3, SW626, SKOV3, and OVCAR3 cells were purchased from ATCC (TCP-1021, HTB-161). KURAMOCHI and OVSAHO cells were gifts from Kevin Elias (Dana-Farber Cancer Institute), and COV362, ES2, JHOS-2, OC314, SNU8, and SNU119 cells were provided in-house. Patient-derived EOC cell lines were generated in-house as previously described (49). Freshly collected ascites were used to obtain a nucleated cell pellet containing immune cells, fibroblasts, mesothelial cells, and cancer cells. These were introduced into tissue culture under 2 different conditions (adherent and nonadherent) and preserved in liquid nitrogen. Low passages of both the adherent and suspension cultured cell lines were used in vitro experiments.

#### PDAC cell lines.

PDAC2, PDAC3, PDAC5, PDAC6, PDAC8, and PDAC9 cells were generated from metastatic ascites fluid of patients with pancreatic adenocarcinoma at the Massachusetts General Hospital as previously described (50). MGH927-1611 cells were gifted from the laboratory of Andrew Liss (Massachusetts General Hospital).

#### CRC and THP-1 cell lines.

All CRC cell lines and the THP-1 cell line were obtained from ATCC. For all in vitro experiments using tumor cell lines, except for LNA transfection, cell lines were grown as tumorspheres under nonadherent conditions in 3D media. For PDAC and CRC cell lines, 3D media contained serum-free RPMI supplemented with 20 μL/mL B27 (Invitrogen/Life Technologies), 20 ng/mL EGF (Invitrogen/Life Technologies), 20 ng/mL bFGF (Invitrogen/Life Technologies), and 1% penicillin/streptomycin (Gibco/Life Technologies). For EOC cell lines, standard base growth medium (see Supplemental Table 1) supplemented with 10% FBS (Thermo Fisher Scientific) and 1% penicillin/streptomycin (Gibco/Life Technologies) was used for 2D adherent cultures, and standard base growth medium without FBS was used for 3D cultures in nonadherent tissue culture dishes. THP-1 cells were maintained with 1% penicillin/streptomycin, 10% FBS, and 0.05 mM β-mercaptoethanol (MilliporeSigma, catalog M3148) in RPMI 1640 medium.

### Drug treatments

IGROV1, OV90, OAW28, and CaOV3 cells were plated at 300,000 cells per well in 6-well ultralow attachment cell culture dishes (MilliporeSigma, catalog CLS3471) in standard growth media. Cells were incubated for 48 to 72 hours to allow tumorsphere formation and then treated with 500 nM 5-azacytidine (MilliporeSigma, catalog A2385), 250 nM Trichostatin A (MilliporeSigma, catalog T1952), or DMSO for vehicle control and incubated for 72 hours. Tumorspheres were then harvested and RNA was isolated for further analyses.

### RNA isolation and RNA-Seq library preparation

Cells (2 × 10^5^ per well) were transferred to 6-well ultralow attachment culture dishes in preferred growth media to allow tumorsphere formation. Tumorspheres were collected on days 3–5 of 3D culture depending on rate of growth. RNA was extracted using the miRNEasy Mini Kit (Qiagen), including the optional column DNAse treatment (Qiagen). In some cases, RNA quality was analyzed using the Bioanalyzer 2100 (Agilent Technologies). To generate libraries for total RNA-Seq, the Clontech-Takara Smarter Stranded Total RNA-Seq kit v2 (catalog 634413) was used according to the manufacturer’s instructions. Pooled libraries were sequenced on an Illumina NextSeq 500 sequencer.

### RNA-Seq data analysis

#### Read alignments.

Reads were trimmed and quality checked using *skewer*. Briefly, ends of the reads were trimmed to remove Ns and bases with quality less than 20. After that, the quality scores of the remaining bases were sorted, and the quality at the 20th percentile was computed. If the quality at the 20th percentile was less than 15, the whole read was discarded. In addition, reads shorter than 40 bases after trimming were discarded. If at least 1 of the reads in the pair failed the quality check and had to be discarded, we discarded the mate as well. Quality filtered reads were mapped using STAR aligner (https://github.com/alexdobin/STAR/releases; commit ID 054b0b807c607c98efd53f817194af090c18a490)and assigned to genes (Gencode annotation) and repeat elements (RepeatMasker annotation) using the *featureCount* function of *Subread* package with the external Ensembl annotation. Unassigned reads were then remapped to the Repbase consensus sequence. Repeat counts from RepeatMasker annotation and Repbase were added together.

#### Count filtering, normalization, and differential expression.

Gene expression in terms of log_2_–counts per million reads (log_2_-CPM) was computed and normalized across samples using the trimmed mean of M values method, as implemented in the calcNormFactors function of edgeR (19). These low-count values (CPM <2), likely due to sequencing errors, were removed before calculating the size factor for each sample. Then, filtered CPM was log_2_ transformed and used in heat-map visualization and Pearson’s correlation analysis. On the heatmap, genes (rows) were scaled by *Z*-score scaling. Heatmaps were generated by the *pheatmap* R statistical programming package. The adjusted *P* value was calculated using Benjamini-Hochberg correction. Differential expression analysis were carried out using *limma* in R; differential expression analysis was performed using *limma* (51).

#### Consensus expression analysis.

Consensus expression of each repeat class was generated using gene set variation analysis (GSVA). The input matrix was normalized log_2_-CPM expression of repeat elements, and a gene list containing predefined gene sets assignment, e.g., SAT, LINE, SINE, ERV, and DNA. The *gsva*() function, which employs the method described by Hänzelmann, Castelo, and Guinney (52), was applied.

### GSEA

GSEA was used to rank all of the genes in the data set based on (a) differential expression calculated by *limma* or (b) the Pearson’s correlation coefficients between consensus expression of repeats and coding genes. To test the gene set significance, an enrichment score was defined as the maximum distance from the middle of the ranked list. Thus, the enrichment score indicated whether the genes contained in a gene set were clustered near the beginning (upregulated/positively correlated) or end (downregulated/negatively correlated) of the list. The GSEA was applied for searching hallmark pathways and was accomplished using the *GSEA* function in *clusterprofiler* (53).

### Immune infiltration analysis

The populations of major types of infiltrating immune cells were evaluated through xCell (R package xCell) (29). The xCell algorithm was used to specifically infer 64 immune and stromal cell types in each sample, based on mRNA expression profiles. The gene length normalized expression profiles of 96 early ovarian cancer samples obtained from patients were prepared and uploaded to the xCell web. Analysis was performed by xCell (*n* = 64) with 1000 permutations, based on the parameter settings. *Q* values for Pearson’s correlation between normalized HSATII expression and different immune infiltration signatures were calculated using function *qvalue* in R with the bootstrap method.

### RNA-ISH

For the detection of HSATII RNA levels, automated RNA-ISH assay was performed using Advanced Cell Diagnostics (ACD) probes against HSATII (ACD 512018) and the RNAscope 2.5 LS Reagent Kit-BROWN from ACD (catalog 322100) on the BondRx 6.0 platform (Leica Biosystems Inc.). 5 μm sections of FFPE tissue (human CRC tissue or cell blocks) were mounted on Surgipath X-tra glass slides, baked for 1 hour at 60°C, and placed on the BOND RX for processing. On the BOND RX, the ACD ISH DAB Protocol was used for staining. The RNA unmasking conditions for the tissue consisted of a 15-minute incubation at 95°C in Bond Epitope Retrieval Solution 2 (Leica Biosystems), followed by 15-minute incubation with Proteinase K. ACD RNAscope probes were used in a final 2-hour probe hybridization step.

The signal was visualized by sequential addition of red substrate for binding to Amp 6 and green substrate for binding to Amp 10, which produced red and green precipitates (dots). The target mRNAs were then visualized using a standard brightfield microscope which showed CYP24A1 signal as Red and FN1 signal as Green.

### RNA-ISH quantification

Slides were imaged using a Leica Aperio CS-O slide scanning microscope at x40 magnification. To determine the relative levels of RNA and protein markers, the images were quantified using Halo software by Indica Labs. The color components for cell nuclei (blue, hematoxylin) and RNA-ISH probe (brown, HSATII) were extracted using color deconvolution. The hematoxylin and probe areas were quantified within representative regions that were annotated by a trained pathologist. Stained RNA-ISH slides were scored according to the fractional area of probe staining in the annotated regions. The fractional probe area was defined as the total probe area divided by total cellular area (sum of hematoxylin and probe areas).

### EV isolations

PDAC and EOC cell lines were grown nonadherently in ULA flasks (Corning) for 5 days prior to EV isolation. The PDAC3 and PDAC6 cells were grown in serum-free medium containing high glucose DMEM with 20 μL/mL B27 (Invitrogen/Life Technologies), 20 ng/mL EGF (Invitrogen/Life Technologies), and 1% penicillin/streptomycin. IGROV1 cells were grown in RPMI medium and OAW28 cells were grown in DMEM/F12, both containing 1% penicillin/streptomycin and 10% FBS. After 3 days in culture, the IGROV1 and OAW28 media was replaced with serum-free media and the cells were cultured for an additional 48 hours. The conditioned medium for each cell line was then centrifuged at 2000*g* for 10 minutes at room temperature to remove larger particles, cells, and debris. Then, 45 μL of media was concentrated, 15 μL at a time, using Amicon Ultra-15 filters (Milipore/Sigma) at 4000*g* at 4°C for approximately 15 minutes per 15 μL. The concentrated media was diluted to 500 μL with filtered PBS and loaded onto 70 nm qEV columns (iZon). An additional 2.35 mL of PBS was added to reach the void volume of 2.85 mL following the manufacturer’s instructions. After allowing the void volume to pass through the column, the EV-rich fractions were eluted with 1.5 mL of PBS and collected. The EVs were quantified using a NanoSight LM10 (Malvern Panalytical) and stored at –80°C.

### EV protein analysis

Concentrated EVs were lysed with 4X Laemmli sample buffer (Bio-Rad, catalog 161-0747) without reducing reagents and boiled for 5 minutes at 95°C. Samples were subjected to SDS-PAGE and then transferred to a PVDF membrane. Membranes were blocked with 3% BSA (MilliporeSigma, catalog A2058) for 1 hour and incubated with primary antibodies overnight at 4°C. Membranes were washed with 1× PBS containing 0.1% tween-20 (MilliporeSigma, catalog P1379) for 10 minutes 3 times and incubated with HRP-conjugated secondary antibodies for 1 to 2 hours at room temperature. After washing, the signal was detected with enhanced chemiluminescence (SuperSignal West Pico PLUS Chemiluminescent Substrate, Thermo Fisher Scientific, catalog 34577), and images were developed using G:BOX (Syngene).

#### Antibodies and concentrations for Western blot analysis.

The following antibodies were used: CD63 (BioLegend, catalog 353039) 1:1000; Flotillin-1 (Cell Signaling, catalog 3253S) 1:1000; CD81 (BioLegend, catalog 349502) 1:1000; CD9 (Cell Signaling, catalog 13174S) 1:1000; HRP-conjugated goat anti-rabbit (Cell Signaling, catalog 7074S); and 1:5000; HRP-conjugated goat anti-mouse (Cell Signaling, catalog 7076S) 1:5000.

#### Coculture of human monocytes and THP-1 cells with tumor-derived EVs.

PBMCs from healthy volunteers were isolated from buffy coats by Ficoll density gradient centrifugation and washed in PBS containing 2% FBS. Following red blood cell lysis, PBMCs were stained with APC-conjugated anti-human CD14 antibody (BD Biosciences, catalog 555399) and sorted on a BD FACSAria II Cell Sorter. CD14^+^ PBMCs were seeded at 15–20,000 cells/well in a 48-well plate in high serum RPMI media containing 20% FBS and 1% penicillin/streptomycin. After 24 hours, the media was replaced with serum-free media and cells were dosed with 1000x EVs isolated from cancer cells and quantified as described above. After 48 hours, the cells were harvested and RNA was extracted using the Qiagen miRNeasy Mini Kit (catalog 217004).

THP-1 cells were plated at 1 × 10^5^ cells/well in ultralow attachment (ULA) 24-well plates (Coring, catalog 3473) without β-mercaptoethanol and FBS. Then, cells were treated with 1 × 10^8^ (for low concentration) and 1 × 10^9^ (for high concentration) particles of EVs derived from OAW28 and IGROV1. After 48 hours, cells were collected, and RNA extraction was performed

### HSATII in vitro transcription and transfection

HSATII containing fragment on chromosome 10 was amplified by PCR with M13 forward and reverse primers as previously described (38) and subjected to in vitro transcription with SP6 RNA polymerase following the MAXIscript SP6 Transcription Kit recommendations (Thermo Fisher Scientific, catalog AM1308). In vitro transcribed HSATII was purified with Megaclear Transcription Clean-Up Kit (Thermo Fisher Scientific, catalog AM1908). Each RNA (500 fmol) was then transfected using jetMESSENGER (Polyplus-transfection, catalog 15001) in accordance with the manufacturer’s instructions. After 48 hours, the cells were harvested and gene expression was analyzed by real-time quantitative PCR.

### Quantitative reverse transcriptase PCR

RNA extraction was performed using the miRNEasy Mini Kit (Qiagen, catalog 217004) according to the manufacturer’s instructions. Total RNA (1 μg) was reverse transcribed using TaqMan Reverse Transcription Reagents (Invitrogen, catalog N8080234). Quantitative reverse transcriptase PCR was performed using the PowerUp SYBR Green Master mix (Applied Biosystems, catalog A25742). The following primers were used: *GAPDH* Forward 5′-ACATCATCCCTGCCTCTACT-3′, Reverse 5′-TCCACCACTGACACGTTG-3′; *DHX58* Forward 5′-ATGACCACCTGGAGATGCCTGA-3′, Reverse 5′-CATTGTAGCGCCTCAGGTGAAG-3′; *IFNB1* Forward 5′-ATGGGAGGCTTGAATACTGC-3′, Reverse 5′-TCATAGATGGTCAATGCGGC-3′; *OAS2* Forward 5′-CCGTTGGTGTTGGCATCTTC-3′, Reverse 5′-GCATTGTCGGCACTTTCCAA-3′; *ISG15* Forward 5′-CTCTGAGCATCCTGGTGAGGAA-3′, Reverse 5′-AAGGTCAGCCAGAACAGGTCGT-3′; *MX1* Forward 5′-TCATAGATGGTCAATGCGGC-3′, Reverse 5′-TCATAGATGGTCAATGCGGC-3′; *MX2* Forward 5′-GCCCTTAGCATGCTCCAGAA-3′, Reverse 5′-ATCGTGCTCTGAACAGTTTGG-3′; *IFI44* Forward 5′-GTGAGGTCTGTTTTCCAAGGGC-3′, Reverse 5′-CGGCAGGTATTTGCCATCTTTCC-3′. Reactions were performed on a QuantStudio 3 (Applied Biosystems) thermocycler. The level of gene expression was calculated based on the 2^–ΔΔCT^ method and normalized to *GAPDH* as an endogenous control. The reference samples were the PBS or no transfection conditions.

### HSATII LNA transfection

OVCAR4, IGROV1, OAW28, and OVSAHO cells were plated at a density of 120,000 cells/well in 6-well tissue-culture treated plates (Corning). OVCAR4 and IGROV1 cells were grown in RPMI medium and OAW28 and OVSAHO cells were grown in DMEM/F12, both containing 1% penicillin/streptomycin and 10% FBS. After 2 days, 500 nM negative control (Scramble) or HSATII custom-designed LNA were transfected into the cells with Lipofectamine 2000 (Invitrogen/Life Technologies) following the manufacturer’s recommendations. One day after transfection, 2 of 3 wells for each experimental condition were trypsinized, counted, and plated for proliferation assays, while the media was changed in the remaining wells for flow cytometry. For proliferation assays, cells were seeded at a density of 1000 cells/well into a 96-well ULA plate (Corning) and quantified using CellTiter-Glo 3D luminescent cell viability assay (Promega) with a SpectraMax microplate reader (Molecular Devices). For flow cytometry, cells were harvested 2 days after transfection and incubated with anti–MHC-I and MHC-II antibodies before quantification on a cytometer.

### Flow cytometric analysis

Following transfection with HSATII-specific or control LNA, IGROV1 and OAW28 (bottom panels) were grown in standard adherent culture for 48 hours. Cells were then trypsinized, washed with PBS, counted, and resuspended at 2.5 × 10^6^ cells/mL in flow cytometry buffer (PBS + 2% FBS). For each condition, 2.5 × 10^5^ cells were stained with PE-conjugated anti-human HLA Class I (R&D Systems, FAB7098P) and APC-conjugated anti-human HLA-DR (Biolegend, 307609) at a concentration of 1:100. After staining, cells were washed and resuspended in flow cytometry buffer with addition of DAPI (Sigma Aldrich, D9542) to a final concentration of 0.1 μg/mL. Single-stained control and experimental samples were run on a Fortessa X-20 flow cytometer (BD Biosciences) and the signal was analyzed using FlowJo software.

### Data availability

All RNA-Seq data have been uploaded to NCBI GEO GSE205430.

### Statistics

For all experiments *P* values were calculated using PRISM9 GraphPad paired 2-tailed *t* test or 2-way ANOVA, unless otherwise noted, and P values of less than 0.05 were considered significant. Kaplan-Meier curve plotted and log-rank analysis performed with GraphPad PRISM9. Additional computational analysis statistics are detailed above. For RNA-Seq data, statistical analyses were performed with R/Rstudio software (version 4.0.3). FDR values of 0.05 were considered significant for GSEA, and *Q* values of 0.1 were used for correlation significance.

### Study approval

Patient-derived cell lines and tumor materials were obtained with approval of Massachusetts General Hospital IRB protocols 2003P001289, 2007P001918, and 2011P001236 and Dana-Farber Harvard Cancer Center (Boston, Massachusetts, USA) IRB protocols 02-240 and 15-015. Briefly, all deidentified ascites samples were collected after obtaining informed consent under a Massachusetts General Hospital IRB-approved protocol (2007P001918). PDAC2, PDAC3, PDAC5, PDAC6, PDAC8, and PDAC9 cell lines were generated from metastatic ascites fluid from patients with pancreatic adenocarcinoma at the Massachusetts General Hospital under a discarded tissue protocol with approval of Massachusetts General Hospital (IRB protocol 2011P001236) and Dana-Farber Harvard Cancer Center (IRB protocol 02-240), as previously described (50). Human ovarian carcinoma tissues for RNA-ISH analysis were obtained from the Dana-Farber Harvard Cancer Center with approval from its IRB (protocol 15-015).

## Author contributions

RLP and SS contributed equally to this work. RLP wrote the original draft, had the initial conception of the project, and obtained funding for this project; therefore, RLP was assigned first authorship. RLP, SS, BDG, and DTT conceived of the study. SS, DP, and A Solovyov provided methodology. SS, MJR, VT, A Solovyov, and BDG provided software. RLP, SS, EY, KHX, LTN, A Solovyov, and BDG provided formal analysis. RLP, EY, IEP, MNF, EB, NKC, ND, ECT, A Szabolcs, ERL, AP, MJR, VT, KHX, and DCR conducted the investigation. DLK, EHS, DP, SLS, VD, JFL, and UAM provided resources. EHS and RLP provided data curation. RLP and DTT wrote the original draft of the manuscript. RLP, SS, BDG, and DTT wrote, reviewed, and edited the manuscript. RLP, SS, EY, MNF, and EB visualized the project. RLP, A. Solovyov, BDG, and DTT provided supervision. RLP, BDG, and DTT provided project administration. RLP, BDG, and DTT acquired funding.

## Supplementary Material

Supplemental data

## Version 1 06/16/2022

In-Press Preview

## Version 2 08/15/2022

Electronic publication

## References

[1] Britten RJ, Kohne DE (1968). Repeated sequences in DNA. Hundreds of thousands of copies of DNA sequences have been incorporated into the genomes of higher organisms. Science.

[2] Lander ES (2001). Initial sequencing and analysis of the human genome. Nature.

[3] Ting DT (2011). Aberrant overexpression of satellite repeats in pancreatic and other epithelial cancers. Science.

[4] Rodic N (2014). Long interspersed element-1 protein expression is a hallmark of many human cancers. Am J Pathol.

[5] Rooney MS (2015). Molecular and genetic properties of tumors associated with local immune cytolytic activity. Cell.

[6] Slotkin RK, Martienssen R (2007). Transposable elements and the epigenetic regulation of the genome. Nat Rev Genet.

[7] Wylie A (2016). p53 genes function to restrain mobile elements. Genes Dev.

[8] Leonova KI (2013). p53 cooperates with DNA methylation and a suicidal interferon response to maintain epigenetic silencing of repeats and noncoding RNAs. Proc Natl Acad Sci U S A.

[9] Chiappinelli KB (2015). Inhibiting DNA methylation causes an interferon response in cancer via dsRNA including endogenous retroviruses. Cell.

[10] Roulois D (2015). DNA-demethylating agents target colorectal cancer cells by inducing viral mimicry by endogenous transcripts. Cell.

[11] Mehdipour P (2020). Epigenetic therapy induces transcription of inverted SINEs and ADAR1 dependency. Nature.

[12] Moufarrij S (2020). Combining DNMT and HDAC6 inhibitors increases anti-tumor immune signaling and decreases tumor burden in ovarian cancer. Sci Rep.

[13] Stone ML (2017). Epigenetic therapy activates type I interferon signaling in murine ovarian cancer to reduce immunosuppression and tumor burden. Proc Natl Acad Sci U S A.

[14] Sheng W (2018). LSD1 ablation stimulates anti-tumor immunity and enables checkpoint blockade. Cell.

[15] Canadas I (2018). Tumor innate immunity primed by specific interferon-stimulated endogenous retroviruses. Nat Med.

[16] Griffin GK (2021). Epigenetic silencing by SETDB1 suppresses tumour intrinsic immunogenicity. Nature.

[17] Tanne A (2015). Distinguishing the immunostimulatory properties of noncoding RNAs expressed in cancer cells. Proc Natl Acad Sci U S A.

[18] Desai N (2017). Diverse repetitive element RNA expression defines epigenetic and immunologic features of colon cancer. JCI Insight.

[19] Solovyov A (2018). Global cancer transcriptome quantifies repeat element polarization between immunotherapy responsive and T cell suppressive classes. Cell Rep.

[20] Panda A (2018). Endogenous retrovirus expression is associated with response to immune checkpoint blockade in clear cell renal cell carcinoma. JCI Insight.

[21] Zapatka M (2020). The landscape of viral associations in human cancers. Nat Genet.

[22] Espinet E (2021). Aggressive PDACs show hypomethylation of repetitive elements and the execution of an intrinsic IFN program linked to a ductal cell of origin. Cancer Discov.

[23] Network CGAR (2011). Integrated genomic analyses of ovarian carcinoma. Nature.

[24] Subramanian A (2005). Gene set enrichment analysis: a knowledge-based approach for interpreting genome-wide expression profiles. Proc Natl Acad Sci U S A.

[25] Liberzon A (2015). The Molecular Signatures Database (MSigDB) hallmark gene set collection. Cell Syst.

[26] Engqvist H (2018). Transcriptomic and genomic profiling of early-stage ovarian carcinomas associated with histotype and overall survival. Oncotarget.

[27] Jayabal P (2021). EZH2 suppresses endogenous retroviruses and an interferon response in cancers. Genes Cancer.

[28] Sun S (2021). Endogenous retrovirus expression activates type-I interferon signaling in an experimental mouse model of mesothelioma development. Cancer Lett.

[29] Aran D (2017). xCell: digitally portraying the tissue cellular heterogeneity landscape. Genome Biol.

[30] Disis ML (2019). Efficacy and safety of avelumab for patients with recurrent or refractory ovarian cancer: phase 1b results from the JAVELIN solid tumor trial. JAMA Oncol.

[31] Matulonis UA (2019). Antitumor activity and safety of pembrolizumab in patients with advanced recurrent ovarian cancer: results from the phase II KEYNOTE-100 study. Ann Oncol.

[32] Konstantinopoulos PA, Cannistra SA (2021). Immune checkpoint inhibitors in ovarian cancer: can we bridge the gap between IMagynation and reality?. J Clin Oncol.

[33] Porter RL, Matulonis UA (2021). Checkpoint blockade: not yet NINJA status in ovarian cancer. J Clin Oncol.

[34] Taki M (2021). Tumor immune microenvironment during epithelial-mesenchymal transition. Clin Cancer Res.

[35] Dongre A (2017). Epithelial-to-mesenchymal transition contributes to immunosuppression in breast carcinomas. Cancer Res.

[36] Pires PRL (2019). Abstract B180: Effects of EMT process under MHC class I and TAP1 gene expression related to antigen presentation. Cancer Immunol Res.

[37] Murakami R (2016). Establishment of a novel histopathological classification of high-grade serous ovarian carcinoma correlated with prognostically distinct gene expression subtypes. Am J Pathol.

[38] Bersani F (2015). Pericentromeric satellite repeat expansions through RNA-derived DNA intermediates in cancer. Proc Natl Acad Sci U S A.

[39] Wang G (2021). The pan-cancer landscape of crosstalk between epithelial-mesenchymal transition and immune evasion relevant to prognosis and immunotherapy response. NPJ Precis Oncol.

[40] Kishikawa T (2016). Quantitation of circulating satellite RNAs in pancreatic cancer patients. JCI Insight.

[42] Goodman AM (2020). MHC-I genotype and tumor mutational burden predict response to immunotherapy. Genome Med.

[43] Lee JH (2020). Transcriptional downregulation of MHC class I and melanoma de- differentiation in resistance to PD-1 inhibition. Nat Commun.

[44] Rodig SJ (2018). MHC proteins confer differential sensitivity to CTLA-4 and PD-1 blockade in untreated metastatic melanoma. Sci Transl Med.

[45] Gu SS (2021). Therapeutically increasing MHC-I expression potentiates immune checkpoint blockade. Cancer Discov.

[46] Zhang L (2003). Intratumoral T cells, recurrence, and survival in epithelial ovarian cancer. N Engl J Med.

[47] Hamanishi J (2021). Nivolumab versus gemcitabine or pegylated liposomal doxorubicin for patients with platinum-resistant ovarian cancer: open-label, randomized trial in Japan (NINJA). J Clin Oncol.

[48] Kulkarni JA (2021). The current landscape of nucleic acid therapeutics. Nat Nanotechnol.

[49] Pépin D (2015). AAV9 delivering a modified human Mullerian inhibiting substance as a gene therapy in patient-derived xenografts of ovarian cancer. Proc Natl Acad Sci U S A.

[50] Indolfi L (2016). A tunable delivery platform to provide local chemotherapy for pancreatic ductal adenocarcinoma. Biomaterials.

[51] Ritchie ME (2015). limma powers differential expression analyses for RNA-sequencing and microarray studies. Nucleic Acids Res.

[52] Hänzelmann S (2013). GSVA: gene set variation analysis for microarray and RNA-seq data. BMC Bioinformatics.

[53] Yu G (2012). clusterProfiler: an R package for comparing biological themes among gene clusters. OMICS.

