# Supplementary material for: Satellite repeat RNA expression in epithelial ovarian cancer associates with a tumor-immunosuppressive phenotype
Source: J Clin Invest. 2022 Aug 15;132(16):e155931. doi: 10.1172/JCI155931 (PMC9374379; doi:10.1172/JCI155931)
Supplement: Supplemental data [file jci-132-155931-s071.pdf]

Satellite repeat RNA expression in epithelial ovarian cancer associates with a tumor immunosuppressive phenotype

Rebecca L. Porter[1,2,3,*], Siyu Sun[4,*], Micayla N. Flores[1], Emily Berzolla[1], Eunae You[1], Ildiko E. Phillips[1], Neelima KC[1], Niyati Desai[1], Eric C. Tai[1], Annamaria Szabolcs[1], Evan Lang[1], Amaya Pankaj[1,5], Michael J. Raabe[1], Vishal Thapar[1], Katherine H. Xu[1], Linda T. Nieman[1], Daniel Rabe[1], David Kolin[6], Elizabeth Stover[3], David Pepin[5], Shannon L. Stott[1], Vikram Deshpande[7], Joyce F. Liu[3], Alexander Solovyov[4], Ursula A. Matulonis[3], Benjamin D. Greenbaum[4,#], David T. Ting[1,2,#]

[1] Mass General Cancer Center, Harvard Medical School; Charlestown, MA, USA.
[2] Department of Medicine, Massachusetts General Hospital, Harvard Medical School; Boston, MA, USA.
[3] Division of Gynecologic Oncology, Dana-Farber Cancer Institute, Boston, MA
[4] Computational Oncology, Department of Epidemiology and Biostatistics, Memorial Sloan Kettering Cancer Center; New York, NY, USA.
[5] Department of Surgery, Massachusetts General Hospital Harvard Medical School; Boston, MA, USA.
[6] Department of Pathology, Brigham and Women's Hospital, Harvard Medical School; Boston, MA, USA.
[7] Department of Pathology, Massachusetts General Hospital, Harvard Medical School; Boston, MA, USA.

* These authors contributed equally to this work

# Address correspondence to
Benjamin Greenbaum, PhD (greenbaB@mskcc.org)
Associate Attending, Computational Oncology
Program Director of Computational Immuno-Oncology
Department of Epidemiology & Biostatistics
Memorial Sloan Kettering Cancer Center
321 E 61st Street, Room 256
New York, NY 10065
(646) 608-7667

David T. Ting, MD (dting1@mgh.harvard.edu)
Associate Clinical Director for Innovation
Massachusetts General Hospital Cancer Center
Associate Professor of Medicine
Harvard Medical School
Building 149, Thirteenth Street, Rm 6-6003
Charlestown, Massachusetts 02129
Tel: 617-240-9402   Fax: 617 724-3676

Supplemental Table 1.

Cell Line	Base Growth Media
CAOV-3	DMEM/NEAA
CAOV-4	DMEM/F12
COV362	DMEM
ES2	RPMI
IGROV1	RPMI
JHOS-2	DMEM/F12
JHOS-4	DMEM/F12
KURAMOCHI	DMEM/F12
OAW28	DMEM/F12
OC314	DMEM/F12
OV90	DMEM/F12
OVCAR3	RPMI
OVCAR4	RPMI
OVCAR8	RPMI
OVKATE	RPMI
OVSAHO	DMEM/F12
PA-1	DMEM
SKOV3	Mod McCoy's 5A
SNU119	RPMI
SNU8	RPMI
SW626	DMEM

A **B**

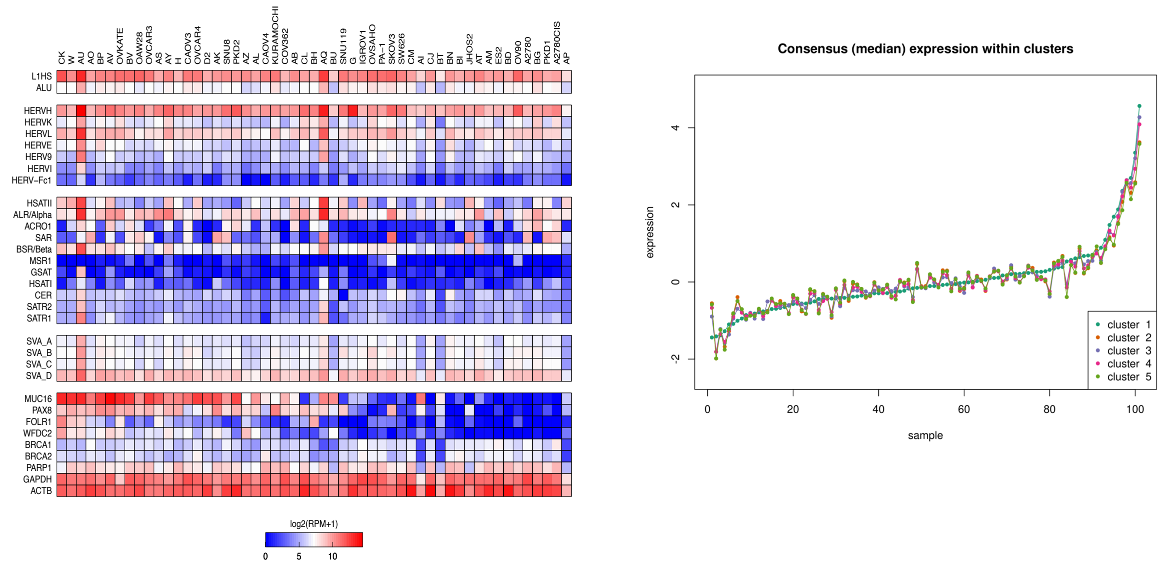

Supplemental Figure 1.
(A) Expression heat map showing log10(RPM) in each cell line/patient sample for a selection of repeat RNAs (including retrotransposons, ERVs and satellites), as well as EOC-associated coding genes and housekeeping genes. **(B)** Median expression of all repeat RNAs within each cluster across all samples.

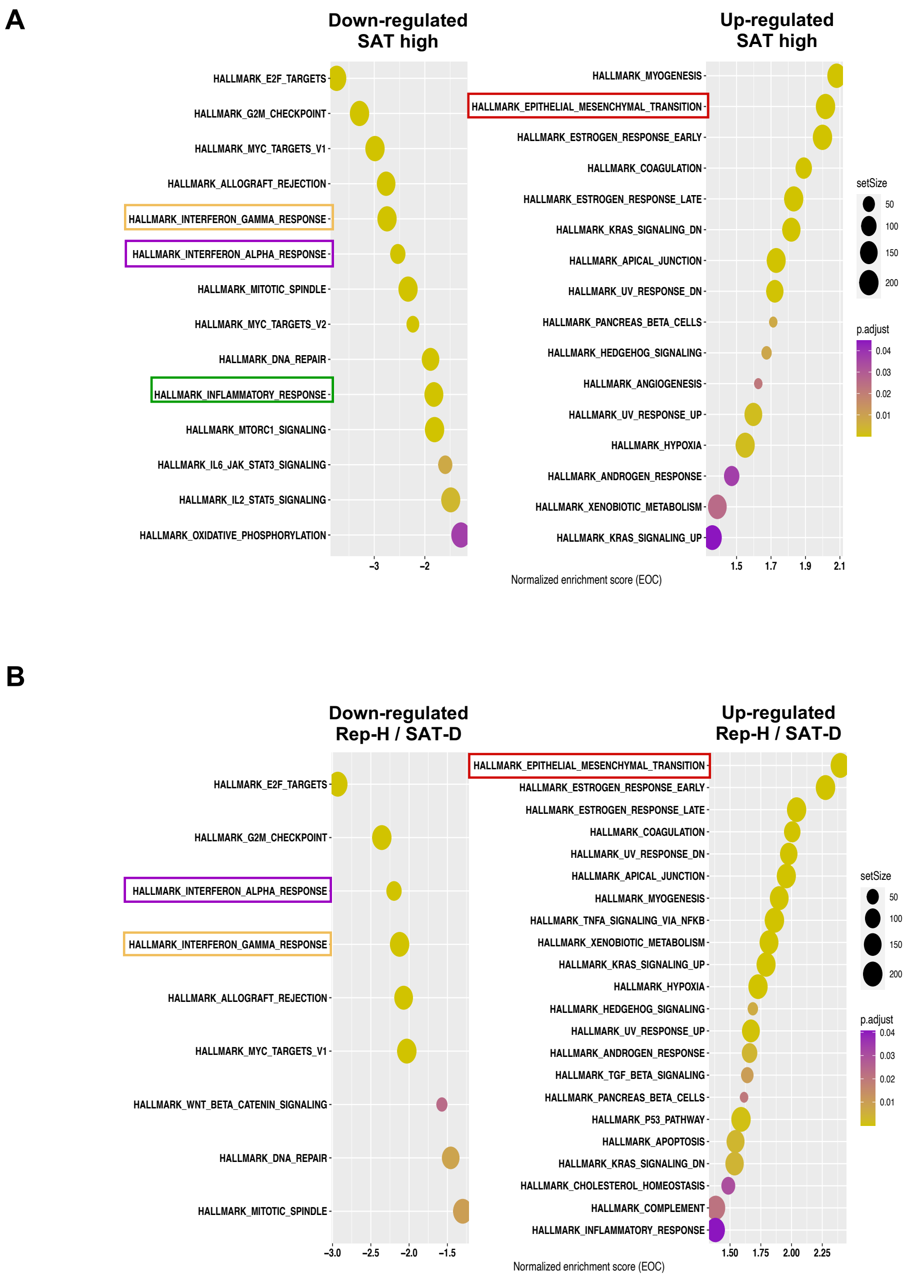

Supplemental Figure 2.

Gene Set Enrichment Analysis (GSEA) of HALLMARK terms of a ranked gene list based on the log_2FC of coding genes for samples in **(A)** SAT-high compared with SAT-low cell lines and **(B)** Rep-H compared with Sat-D clusters. Circle size represents gene set size and color represents adjusted p-value as shown in legend.

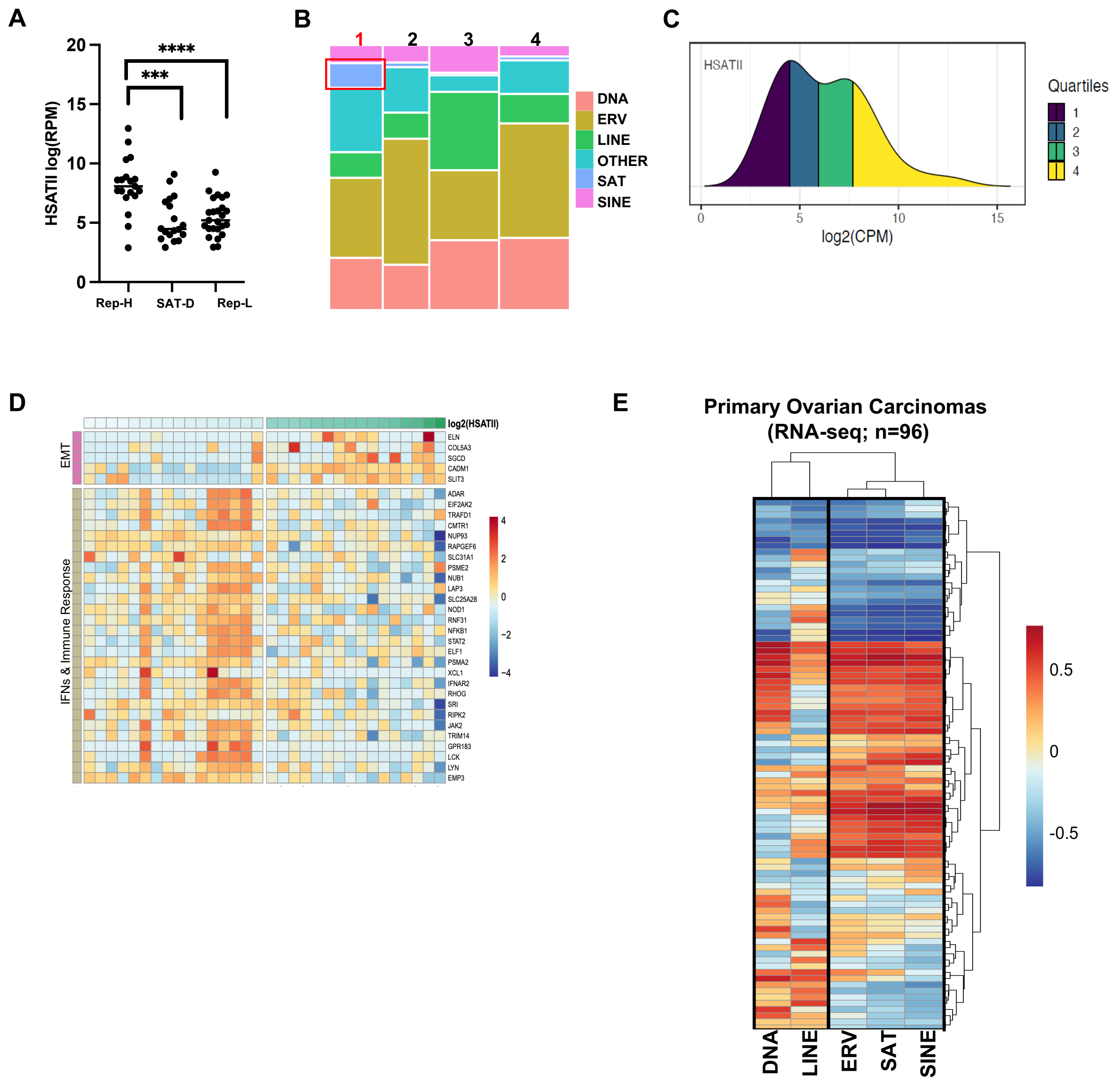

Supplemental Figure 3.
(A) HSATII RNA expression in cell lines within each repeat cluster, expressed as $\log_2$(reads per million). **(B)** Mosaic plot demonstrating relative repeat element subclass composition of each consensus cluster from Fig. 4C. **(C)** Expression of HSATII, graphed as log2(copies per million reads) across all cell lines, separated in quartiles. **(D)** Expression heatmap of differentially expressed genes related to the EMT (top) and IFN and immune response pathways (bottom) across HSATII-high and HSATII-low cell lines expressed as scaled $\log_2$(normalized counts per million). **(E)** Hierarchical clustering of consensus expression calculated for each repeat subclass in early-stage ovarian carcinomas.

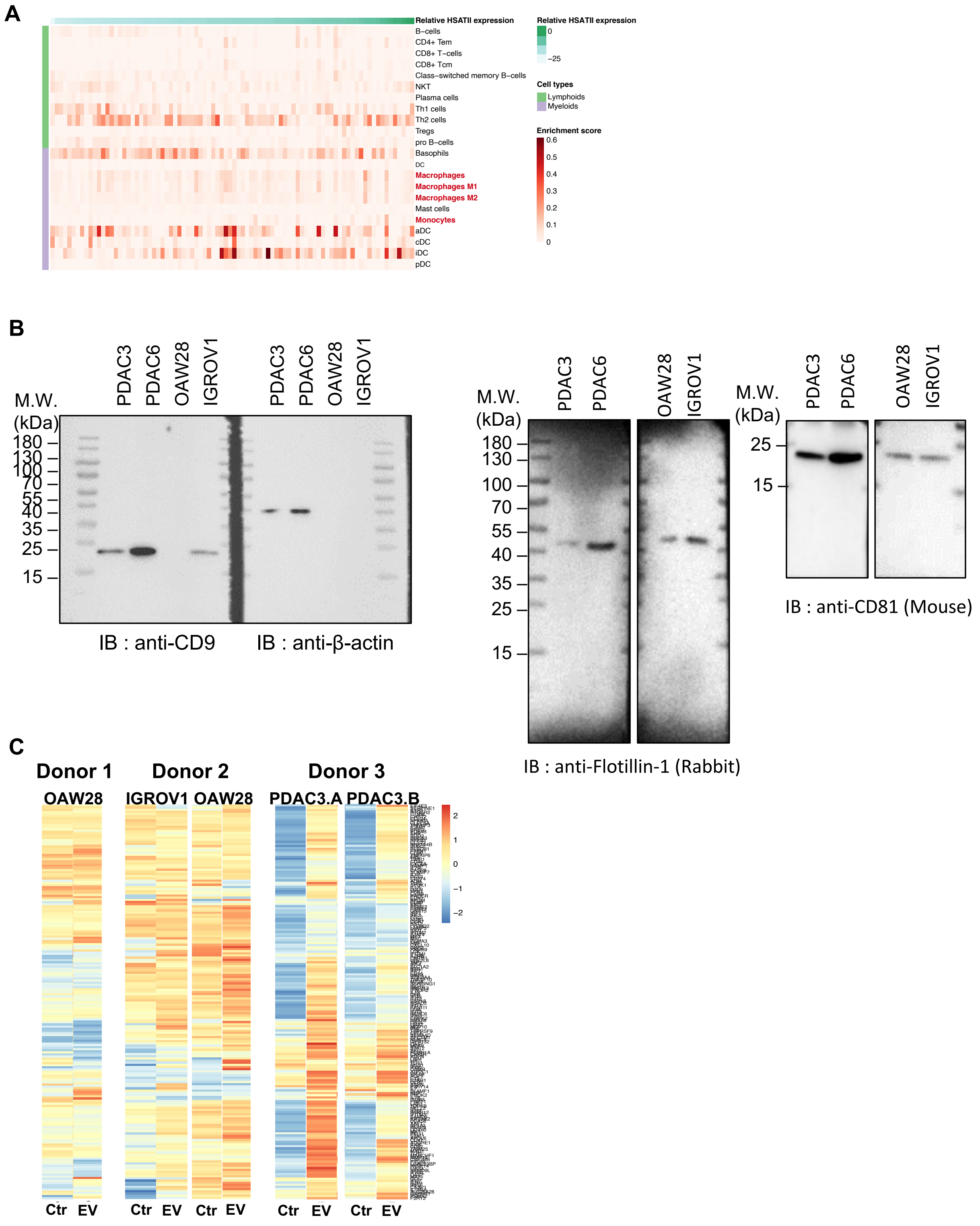

Supplemental Figure 4.
(A) Heatmap of the relative frequency (Enrichment score) of immune cell types from Lymphoids & Myeloids in early-stage ovarian carcinoma tumor samples as identified by the “xCell” algorithm. The corresponding normalized HSATII expression in each sample is plotted on the top. **(B)** Western blots demonstrating protein levels of common EV surface proteins CD9, Flotillin-1 and CD81 on isolated EOC and PDAC cell line-derived EVs. **(C)** Expression heatmap of genes (z-score scaled by row) related to activation of innate immune interferon responses in primary human $CD14^{+}$ peripheral blood mononuclear cells (PBMCs). EV and Ctr indicate whether the samples have been (EV) or not been (Ctr) exposure to EOC or PDAC cell line derived EVs.

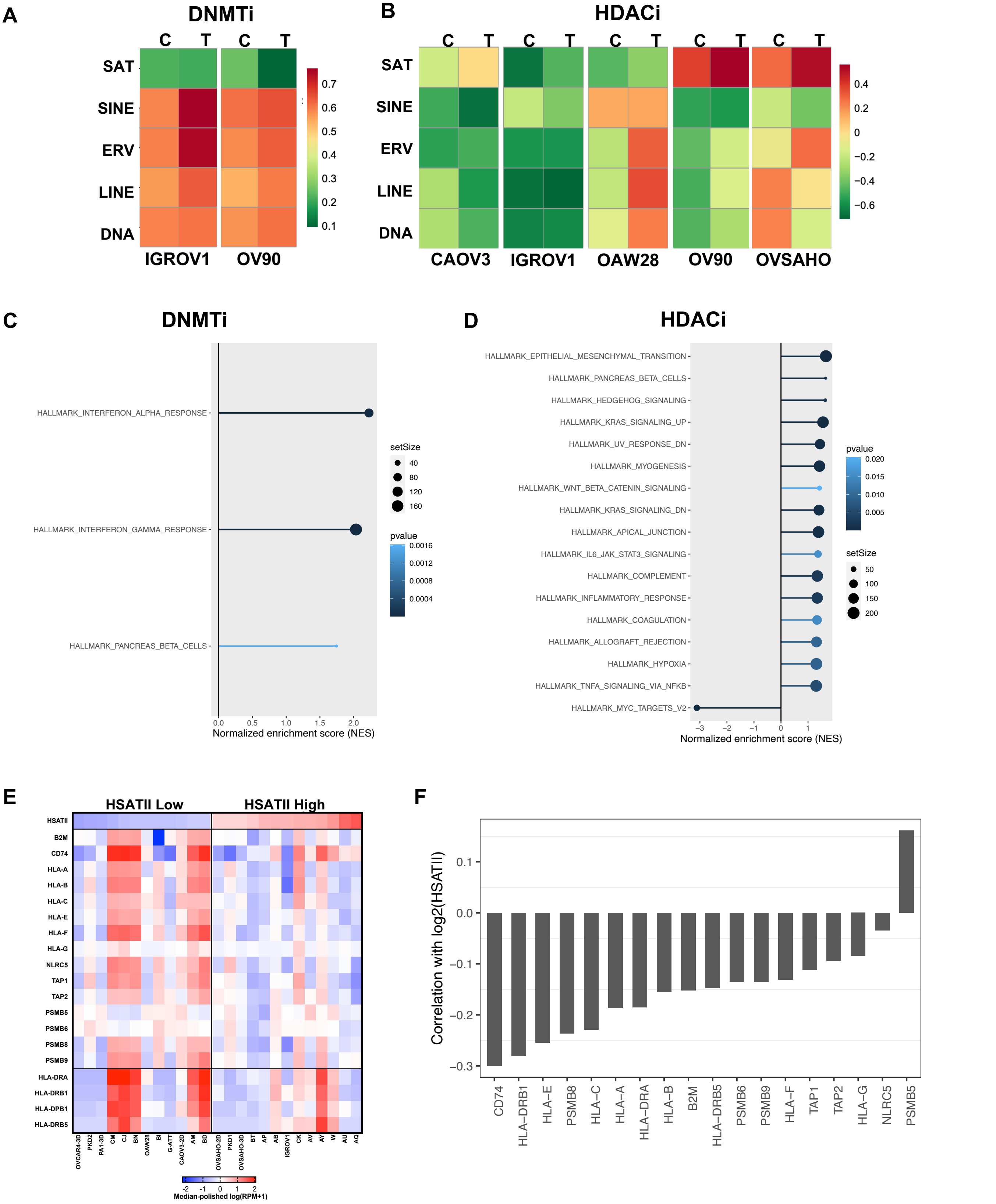

Supplemental Figure 5.
(**A-B**) Heat maps of repeat RNA subclass consensus expression estimated using GSVA (see Methods) in individual EOC cell lines treated with 500 nM 5-azacytidine (DNMT3 inhibitor; T) or 250 nM Trichostatin A (HDAC inhibitor; T) compared with vehicle control (C) for 72 hours. (**C-D**) GSEA of HALLMARK terms based on the log2FC of coding genes in EOC cell lines treated with DNMT3 inhibitor or HDAC inhibitor over corresponding vehicle control. (**E**) Expression heatmap depicting expression of MHC-Class I and II genes in HSATII-low and HSATII-high EOC cell lines, plotted as median-polished log(RPM+1). (**F**) Pearson correlation coefficients between HSATII expression and the expression of MHC-Class I genes and PD-L1 in early-ovarian tumor samples.
